# Lower dose zinc for childhood diarrhea: a randomized, multicenter trial

**DOI:** 10.1056/NEJMoa1915905

**Published:** 2020-09-24

**Authors:** The Zinc Therapeutic Dose Trial (ZTDT) Research Group, Usha Dhingra, Rodrick Kisenge, Christopher R. Sudfeld, Pratibha Dhingra, Sarah Somji, Arup Dutta, Mohamed Bakari, Saikat Deb, Prabhabati Devi, Enju Liu, Aishwarya Chauhan, Jitendra Kumar, Om Prakash Semwal, Said Aboud, Rajiv Bahl, Per Ashorn, Jonathon Simon, Christopher P. Duggan, Sunil Sazawal, Karim Manji

**Affiliations:** 1 Center for Public Health Kinetics (CPHK), New Delhi, India; 2 Muhimbili University of Health and Allied Sciences (MUHAS), Dar es Salaam, Tanzania; 3 Boston Children’s Hospital, Boston, USA; 4 Harvard T.H. Chan School of Public Health, Boston, USA; 5 World Health Organization, Geneva, Switzerland

**Keywords:** zinc, diarrhea, vomiting, child health

## Abstract

**Background:**

The WHO recommends 20 mg/day of supplemental zinc for children with acute diarrhea for 10-14 days; in previous trials this dosage improved diarrhea but increased vomiting.

**Methods:**

We randomly assigned 4500 children ages 6 to 59 months in India and Tanzania with acute diarrhea to one of three arms (5, 10 or 20 mg zinc sulfate for 14 days). The three primary outcomes were diarrhea duration >5 days and mean number of stools (tested for non-inferiority), and vomiting within 30 minutes of zinc administration (tested for superiority).

**Results:**

The proportion of children with diarrhea duration >5 days was 6.5%, 7.7%, 7.2% in the 20 mg, 10 mg and 5 mg groups, respectively. The difference between 20 mg and 10 mg dosages was 1.2% (upper bound of one-sided 98.75% CI 3.6%) and between 20 mg and 5 mg was 0.7% (upper bound 3.0%), both below the 4% noninferiority margin (4%). The mean number of loose stools was 10.7, 10.9, and 10.8 in the 20 mg, 10 mg and 5 mg groups, respectively. The differences between 20 mg and 10 mg groups was 0.3 (upper bound 1.1), and between 20 mg and 5 mg group, 0.1 (upper bound 0.9), both below the noninferiority margin (2 stools). Vomiting within 30 minutes of zinc administration occurred in 19.3%, 15.6%, 13.7%, respectively, and was significantly lower in the 10 mg (relative risk 0.81, 97.5% CI 0.67 to 0.96) and 5 mg (0.71, 97.5% CI 0.59 to 0.86) groups. Both dosages also reduced vomiting after 30 minutes of dosing.

**Conclusion:**

Compared with the standard 20 mg dosage of zinc, lower dosages had noninferior efficacy for diarrhea and reduced vomiting in children with diarrhea. NCT03078842.

## Introduction

Although we have witnessed a 90% decline in diarrhea deaths over the past four decades, diarrheal diseases remain a major public health problem. In 2018, approximately 500,000 children died from diarrhea. Most of these deaths could be avoided if children received high-quality management using the World Health Organization (WHO) and the United Nations Children’s Fund (UNICEF) recommended care, which includes oral rehydration solutions and supplemental zinc.^1^ WHO and UNICEF currently recommend 20 mg zinc daily supplementation for 10–14 days in addition to oral rehydration solutions for the management of acute diarrhea in children.^2^ This recommendation is based on studies demonstrating that supplemental zinc results in a shorter duration of diarrhea, reduces number of stools and stool output, reduces the risk of persistent diarrhea, and may reduce the risk of subsequent illness episodes and increase weight gain.^3-8^

The currently recommended dose was based on assumptions of increased zinc losses during diarrhea and the need for additional zinc above the recommended dietary allowance (RDA) for mucosal regeneration.^9^ Replication studies generally used the 20 mg zinc dose without any further dose-ranging studies.This dose substantially exceeds the RDA for zinc in infancy and early childhood (2-5 mg/day).^10^

Zinc given orally can cause vomiting due to its strong metallic taste in saliva and gastric irritability; both of which are dose-dependent.^11^ In a meta-analysis of 11 acute diarrhea trials (n=4438)^4^, subjects who received supplemental zinc were significantly more likely to vomit with the initial dose than with placebo (12.7% vs. 7.6%; RR: 1.55; 95% CI: 1.30 to 1.84). In another review^12^, the risk of vomiting was significantly higher with zinc supplementation in children older than age 6 months (risk ratio 1.57, 95% CI 1.32 to 1.86; 2605 children, 6 trials).

Lower doses of zinc, provided they are equally effective, might have the advantage of reducing associated vomiting. We therefore performed a randomized, double-blind controlled trial comparing two lower doses of zinc with the current recommended dose in low- and middle-income country settings. We hypothesized that lower zinc doses (5 or 10 mg/day) compared with standard zinc dose (20 mg/day) would be non-inferior with respect to diarrhea treatment efficacy, but superior with respect to side effect profile (i.e., vomiting).

## Methods

The trial’s methodology has been published and the protocol is available with this article at nejm.org.^13^ In brief, the Zinc Therapeutic Dose Trial (ZTDT) was an individually randomized, parallel group, double-blind, controlled trial of three doses of supplemental zinc among children ages 6 to 59 months in India and Tanzania (ClinicalTrials.gov identifier NCT03078842).

Study personnel screened all children presenting with illness to outpatient health facilities to detect diarrhea. Subjects were children with acute diarrhea for fewer than 72 hours (defined as three or more loose or watery stools per 24 hours) or dysentery (defined as acute diarrhea with visible blood in the stool) whose families were likely to stay within the study area for at least 2 months after enrollment and whose caretakers provided written informed consent. Excluded were children with any one of the following: severe acute malnutrition (weight for length/height Z score (WHZ) of <-3 or presence of edema), severe dehydration that could not be corrected within 4 to 6 hours, severe pneumonia (characterized by presence of fast breathing or chest in-drawing and any of the following danger signs: inability to breastfeed or drink, lethargy or unconsciousness, convulsions, or vomiting everything), clinically-suspected bacterial sepsis, rapid diagnostic test-confirmed malaria, or other severe illness. In addition, children who were previously enrolled in the study, whose siblings were currently enrolled in the study, who were currently enrolled in another trial, or who had used zinc supplements during the three days preceding study enrollment were excluded.

Subjects were enrolled from peri-urban outpatient health facilities in two countries (India and Tanzania). In India, recruitment took place at Sangam Vihar, a resettlement colony near South Delhi and in Harsh Vihar, a semi-urban locality in Northeast Delhi. In Tanzania, recruitment took place in the outpatient clinics of Temeke District Hospital and Mbagala Rangi Tatu hospital, and the Mbagala Round Table health center, all in Dar es Salaam.

### Interventions

Children were randomly allocated to receive one of three zinc sulfate regimens; 5 mg, 10 mg or 20 mg (each taken once daily for 14 days). Study staff instructed caretakers to dissolve the dispersible tablet in 5-10 mL of water or breastmilk immediately before administration. After an initial dose provided under direct supervision of study staff on the day of enrollment, children received the zinc supplementation from their caretakers for 14 days total. Study regimen was manufactured by Laboratoires Pharmaceutiques Rodael S.A.S, France and shipped to WHO for randomization and labelling before shipment to recruitment sites.

### Outcomes

The primary efficacy outcomes were 1) the proportion of enrolled children who had duration of diarrhea of >5 days, and 2) the number of loose or watery stools during the diarrhea episode after randomization. We defined diarrhea as the occurrence of 3 or more loose or watery stools per day. The last day of diarrhea was defined as the day prior to two diarrhea-free days. The duration of the diarrhea episode was defined as the number of days between randomization and the first day without diarrhea. The primary side effect outcome was the occurrence of vomiting within 30 minutes of administration of the zinc supplement over the 14-day course of treatment. This dose-related vomiting was measured by direct observation on day 1 and subsequently by caretaker report of vomiting recorded on a daily diary. Caretakers also used the daily diary to record compliance with the intervention, numbers of stools, and non-dose-related vomiting (>30 minutes after dose administration). This diary was reviewed in person by trained field workers at periodic home or clinic visits on days 3, 5, 7, 10 and 15. (The day 5 and 10 visits were made by phone in Tanzania.)

Secondary outcomes included the percentage of children who had diarrhea >3 days, adherence to zinc treatment (number of tablets consumed, reported favorable child acceptability), plasma zinc levels on days 1, 3, 7, 15, 21 and 30, illness in the 60-day period following initiation of treatment (diarrhea, fever or respiratory symptoms), and growth in the 60-day period following initiation of treatment (changes in weight, length, mid-upper arm circumference).

### Laboratory methods

Venous blood samples (3-5 ml) were collected in trace element-free syringe by trained technicians, transferred to zinc-free heparin tubes, spun down at 15 minutes after blood collection and aliquots transferred into trace element-free storage tubes at −80°C until analysis.^14^ Plasma samples were analyzed for zinc status via atomic absorption spectrometry (AAS 400-Perkin Elmer, USA) at the Center for Public Health Kinetics micronutrient research laboratory at Subharti Medical College, Meerut, India. Samples were obtained from a randomly selected 1/3 of subjects at baseline.

### Sample size

Considering a 1:1:1 random allocation, significance level of 0.05 (one-sided for diarrhea duration and mean stool number output non-inferiority tests, and two-sided for vomiting superiority tests), 90% power, and a 5% loss to follow-up rate, 4500 subjects were needed.^13^ No interim analyses by study personnel were planned.

For the efficacy outcome duration of diarrhea >5 days, we assumed a risk of 16% in the standard dose arm^6,15^ and chose a 4% absolute risk difference non-inferiority margin since it represented the difference between zinc supplementation and placebo in a previous trial^15^. For the efficacy outcome number of loose/watery stools after enrollment, we assumed a mean (SD) stool number of 10 (9) in the standard dose arm^15^ and chose a non-inferiority margin of 2 stools since it roughly corresponded to the reduction in stool output noted in the literature^4,6^. For our primary side effect profile outcome, we hypothesized a 25% relative risk reduction in the occurrence of vomiting (from 20% of children to 15%).

### Blinding and randomization

The allocation ratio was 1:1:1 and was stratified by country (India and Tanzania) and age (< 24 months and ≥ 24 months). All three study tablets (5, 10 and 20 mg) were identical in appearance, taste and smell and were packaged and distributed in identical blister packs. Each pack was labelled with a unique subject identification number from a computer-generated permuted block randomization list with variable block size generated off-site by a non-study statistician in WHO, Geneva. Four lists were created corresponding to the four strata. To maintain allocation concealment, prelabeled blister packs were shipped to the study sites, and enrolled subjects were provided the next sequentially numbered regimen in the stratum. Study staff screened and enrolled subjects. Other clinical staff were responsible for distributing study regimen to the caretaker of the subject and observing the first dose. The randomization code linking the unique ID number with treatment arm was broken only after all primary outcomes were analyzed in a blinded fashion. All enrolled children, their caretakers, research staff, any treating providers, and the study statisticians were thus blinded to study arm.

### Statistical methods

All primary analyses used the intention-to-treat (ITT) principle, and sensitivity analyses were also performed using a per-protocol (PP) approach for non-inferiority outcomes.^16^ The ITT analysis included all randomized patients with assessable data, and the PP analysis included subjects documented to have taken all zinc supplements for the first 5 days after randomization. Missing data for primary outcomes was negligible.

The first efficacy outcome was the proportion of children with diarrhea duration >5 days. Children who did not have resolution of diarrhea and were lost to follow-up, died, or who withdrew before day 5 were not included in this analysis. The risk differences for children with diarrhea duration >5 days between the lower doses and the standard dose were estimated and a one-sided 95% confidence interval and comparing it to a predefined non-inferiority margin of 4%. Binomial generalized estimating equations (GEE) with identity link were used to estimate the risk difference, and log link were used to estimate risk ratio.^17,18^ We constructed a Kaplan-Meir curve for time to recovery of initial diarrhea episode by randomized group and used the log-rank test to test the differences in the incidence rate of recovery in the lower zinc dose groups compared to the 20 mg group. In addition, Cox proportional hazard models were used to estimate hazard ratios and their 95% confidence intervals for recovery from the initial diarrhea for the lower dose zinc arms compared to the 20 mg group.

The second primary efficacy outcome was the total number of loose or watery stools during the presenting diarrhea episode. Mean differences in the total number of loose or watery stools between the lower doses and the standard dose were estimated and a 95% one-sided confidence interval was compared with a predefined non-inferiority margin of 2 loose or watery stools. Analyses of covariance were used to estimate the mean differences and corresponding 95% confidence intervals.

The primary side effect outcome was vomiting within 30 minutes of administration of each dose of the trial regimen. Log-binomial regressions were used to assess relative risks and 95% confidence intervals of ever vomiting during the 14-day study period comparing the lower doses with the standard dose. Generalized estimating equations (GEE) with the log link, binomial distribution and exchangeable correlation matrix were also used to assess the relative risk of vomiting comparing the lower doses with the standard dose. The exchangeable working covariance matrix and robust estimators of the variances were used to construct 95% confidence intervals.

We performed post hoc Bonferroni correction for the primary outcome efficacy outcomes by providing 98.75% confidence intervals instead of 95% confidence intervals to account for multiple comparisons (duration of diarrhea and number of stools, 10 mg vs. 20 mg and 5 mg vs. 20 mg). Similarly correction was applied to safety primary outcome (vomiting) by providing 97.5% confidence intervals to account for multiple comparisons (10 mg vs. 20 mg and 5 mg vs. 20 mg). In addition, statistical significance of primary efficacy outcome were assessed with a P value <0.0125. A similar correction was applied to the primary safety outcome (vomiting within 30 minutes) by providing 97.5% confidence intervals to account for multiple comparisons (10 mg vs. 20 mg and 5 mg vs. 20 mg) and statistical significance of the safety outcome was assessed with a P value <0.025 .

### Secondary outcomes

We used a linear mixed-effects model to assess differences in plasma zinc response for the 5 mg and 10 mg arms compared to the 20 mg arm. The model included treatment arm, time of blood draw and an interaction term between treatment arm and time of blood draw. Time of blood draw was binned in accordance with the sampling schedule: baseline, Day 3 (3-5), Day 7 (6-11), Day 15 (12-18), Day 21 (19-25) and Day 30 (26-45). The model accounted for within-subject correlation with an unstructured correlation matrix to allow for flexibility due to the complex sampling design. The unstructured correlation matrix also resulted in the lower Akaike information criterion values (AIC) as compared to other correlation structures, which indicated better model fit. Mean differences and 95% confidence intervals were estimated for the 5 mg group and 10 mg group compared to the 20 mg group for each time point.

Relative risks or mean differences with their 95% confidence intervals were calculated for other secondary outcomes. For the secondary outcomes, unadjusted 95% confidence intervals are provided for the differences between the groups; hence they cannot be used to infer effects.

### Subgroup analyses to explore effect modification

Although the trial was not designed to draw conclusions about modification of treatment effects, the following prespecified exploratory subgroup analyses based on baseline characteristics were performed: study site (India/Tanzania), age (<12 months, >12 months), sex (male/female), rotavirus immunization (no/yes), dysentery (no/yes), dehydration (no/yes), temperature >38^o^C (no/yes), respiratory rate >40 (no/yes), recent antibiotic use (no/yes), breastfed (no/yes), stunted (no/yes), underweight (no/yes), wasted (no/yes), mid-upper arm circumference <125 mm (no/yes), family wealth <median (no/yes), maternal education <8 years (no/yes), improved water (no/yes), improved sanitation (no/yes), and plasma zinc concentrations (<65 vs. ≥65 μg/dL). Stratified risk differences and relative risks and their 95% CIs are presented.

Statistical analyses were performed with STATA 14 (Stata Corp., College Station, Texas, USA), SPSS 25 (SPSS Inc., Chicago, Illinois, USA and SAS, version 9.4 (SAS Institute, Cary, NC).

### Standard of care and ethics

All enrolled children received standard of care for diarrhea management per WHO/UNICEF Integrated Management of Childhood Illness (IMCI) diarrhea case management guidelines^19^, and Indian and Tanzanian Ministry of Health guidelines. All caretakers signed written informed consent for their child’s participation. The protocol was approved by the WHO Ethics Review Committee (reference ERC.0002738), Boston Children’s Hospital IRB (reference IRB-P00024269), Tanzania Food and Drug Authority (reference TFDA0016/CTR/0015/03), the Tanzanian National Institute of Medical Research (reference NIMR/HQ/R.8a/Vol.IX/2333) the Muhimbili University of Health and Allied Sciences, Dar es Salaam (reference 2016-10-31/AEC/Vol.XI/314) and the Institutional Ethics Committee of Subharti Medical College & Hospital, Meerut, UP, India (reference SMC/EC/2016/84). The ZTDT Data Safety and Monitoring Board met twice during the trial to review patterns of SAEs and other safety outcomes. In June 2018 they conducted an Interim Efficacy and Safety analysis and did not recommend any changes in the trial’s conduct.

## Results

### Patients

**Figure 1:**
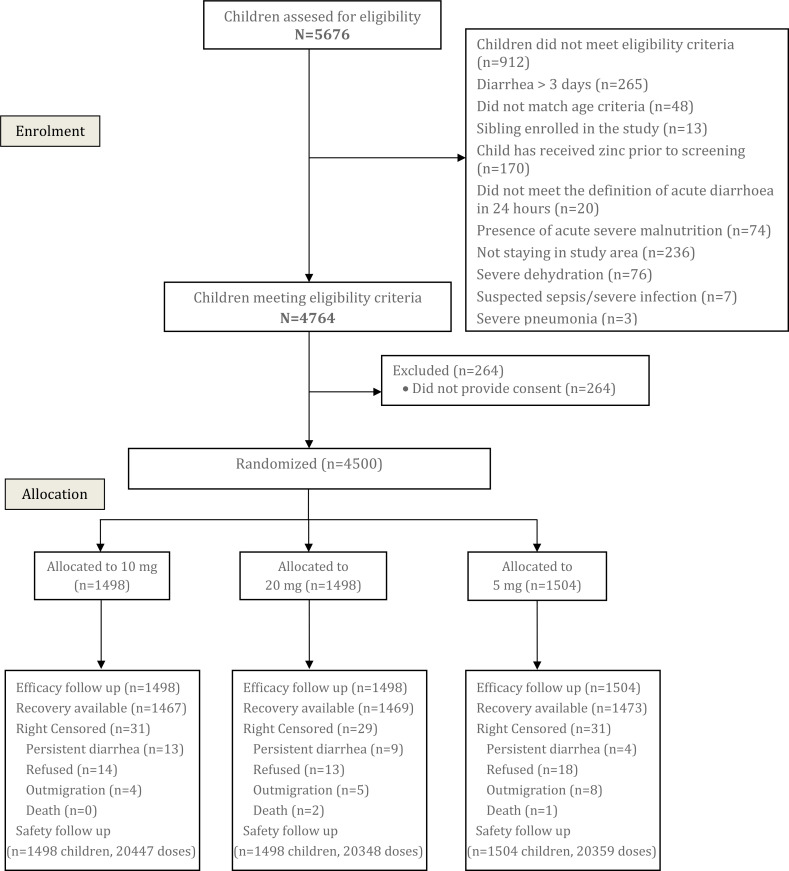
Study Flow Diagram

From January 2017 to February 2019, 5676 children with diarrhea were screened for eligibility and 4500 children were enrolled, of whom 1/3^rd^ were randomized to each of the three arms (Figure 1). Follow-up was achieved for ~98% of enrolled children for the primary outcomes. The characteristics of the children (Table 1) were generally well-balanced at baseline among the three groups: household characteristics were comparable and mean (SD) age of the children was 23.0 (14.7), 22.7 (14.6) and 23.2 (15.3) months in the 5, 10 and 20 mg arms, respectively. The majority of children had no dehydration upon presentation, and the mean number of loose or watery stools in the 24 hours before enrollment was 5.7 in all three arms. Data on baseline characteristics by study site are provided in Supplementary Tables A and B.

**Table 1:** Baseline characteristics of the trial population stratified by zinc supplementation dose (n=4500)

	5 mg (N=1504) Mean ± SD or n (%)	10 mg (N=1498) Mean ± SD or n (%)	20 mg (N=1498) Mean ± SD or n (%)
*Study site*			
India	753 (50.1)	749 (50.0)	748 (49.9)
Tanzania			
	751 (49.9)	749 (50.0)	750 (50.1)
*Maternal and household characteristics*			
Maternal age, years	26.7 ± 5.0	26.8 ± 5.0	26.9 ± 5.0
Maternal education, years	7.3 ± 4.2	7.1 ± 4.1	7.3 ± 4.0
No maternal education	257 (17.3)	254 (17.1)	230 (15.5)
Improved water	1485 (99.1)	1485 (99.3)	1485 (99.3)
Improved sanitation	1486 (99.1)	1488 (99.5)	1489 (99.5)
Household wealth above median^1^	760 (50.7)	738 (49.3)	772 (51.6)
			
*Child characteristics*			
Age at randomization (months)	23.0 ± 14.8	22.7 ±14.6	23.2 ± 15.3
Age at randomization			
6 to <12 months	412 (27.4)	410 (27.4)	434 (29.0)
12 to <24 months	499 (33.2)	502 (33.5)	476 (31.8)
24 to <60 months	593 (39.4)	586 (39.1)	588 (39.3)
Female	711 (47.3)	725 (48.4)	719 (48.0)
Breastfeeding day prior to enrollment	876 (58.4)	857 (57.3)	853 (57.1)
Rotavirus vaccination^2^	749 (49.8)	741 (49.5)	748 (50.0)
Duration of diarrhea before enrollment			
≤ 24 hours	58 (3.9)	48 (3.2)	59 (3.9)
25 to 48 hours	1259 (83.7)	1224 (81.7)	1231 (82.2)
49 to <72 hours	187 (12.4)	226 (15.1)	208 (13.9)
Number of loose or watery stools in the 24 hours before enrollment	5.7 ± 2.0	5.7 ± 2.1	5.7 ± 2.1
Dysentery	54 (3.6)	62 (4.1)	51 (3.4)
Some dehydration	32 (2.1)	11 (0.7)	13 (0.9)
Axillary temperature >38°C	48 (3.2)	40 (2.7)	34 (2.3)
Cough or difficulty breathing	405 (26.9)	438 (29.2)	424 (28.3)
Observed respiratory rate >40 bpm	86 (5.7)	84 (5.6)	85 (5.7)
Prior antibiotic use	32 (2.1)	38 (2.5)	26 (1.7)
Height (cm)	79.9 ± 11.0	79.7 ± 11.0	80.1 ± 11.7
Weight (kg)	10.0 ± 2.5	9.9 ± 2.5	10.0 ± 2.7
MUAC (cm)	14.1 ± 1.2	14.0 ± 1.2	14.1 ± 1.2
Mean LAZ/HAZ	-1.3 ± 1.2	-1.3 ± 1.1	-1.3 ± 1.2
Mean WLZ/WHZ	-0.7 ± 1.0	-0.7 ± 1.0	-0.7 ± 1.0
Mean WAZ	-1.2 ± 1.1	-1.2 ± 1.0	-1.2 ± 1.1
Mean MUACZ	-0.8 ± 1.0	-0.8 ± 1.0	-0.8 ± 1.0
Stunted (LAZ/HAZ < -2)	424 (28.2)	382 (25.5)	382 (25.5)
Wasted (WLZ/WHZ < -2	131 (8.7)	135 (9.0)	141 (9.4)
Underweight (WAZ < -2)	353 (23.5)	348 (23.2)	333 (22.2)
Zinc dose (mg/kg body weight)	0.53 ± 0.13	1.07 ± 0.25	2.14 ± 0.54
Plasma zinc concentration (μg/dL)^3^	71.5 ± 23.4	74.9 ± 23.9	74.0 ± 27.1
Plasma zinc concentration <65 μg/dL^3^	175 (40.1)	143 (33.1)	174 (39.5)

1 Country-specific household wealth index was constructed using a principal component analysis of household ownership, household assets, drinking water source, and sanitation

2 More than 99% of Tanzanian children received at least one rotavirus vaccination while <1% of children in India received at least one rotavirus vaccination

3 Plasma zinc concentrations were assessed in a random sample of 33% of participants at baseline at each site

### Primary Outcomes

Intention to treat analyses (Table 2) showed that the proportion of children with diarrhea >5 days was similar across all three zinc arms (6.5% in the standard 20 mg group, 7.7% in the 10 mg group and 7.2% in the 5 mg group). Compared with those assigned to 20 mg, children assigned to 10 mg of zinc had a risk difference of 1.2% (upper bound of one-sided 98.75% CI 3.6%) while children assigned to 5 mg of zinc had a risk difference of 0.7% (upper bound of one-sided 98.75% CI 3.0%). In both cases, the upper bound of 98.75% CI was less than the pre-defined 4% non-inferiority margin. Results from per protocol analyses were similar to those from the ITT analyses. A Kaplan-Meir curve for time to recovery of the initial diarrhea episode by randomized group is presented in Supplemental Figure C.

**Table 2:** Effect of zinc supplementation dose on diarrhea and vomiting in children with acute diarrhea.

		5 mg	10 mg	20 mg
Diarrhea Related Outcomes				
Diarrhea > 5 days (Intention to treat analysis)	n/N (%)	106 /1480 (7.2%)	114 / 1480 (7.7%)	96 / 1479 (6.5%)
	Risk difference and upper bound of one-sided 98.75% CI^1^	0.7% (2.8%)	1.2% (3.3%)	Ref
	Non-inferiority one-sided p-value (+4% margin)	<0.001	0.002	Ref
Diarrhea > 5 days (Per protocol analysis)	n/N (%)	102 / 1431 (7.1%)	109 /1437 (7.6%)	93 /1440 (6.5%)
	Risk difference and upper bound of one-sided 98.75% CI	0.7% (2.8%)	1.1% (3.3%)	Ref.
Total loose or watery stools after enrolment (Intention to treat analysis)	N	1496	1488	1490
	Mean + SD	10.8 ± 8.9	10.9 ± 9.2	10.7 ± 8.7
	Mean difference and upper bound of one-sided 98.75% CI^1^	0.1 (0.8)	0.3 (1.0)	Ref
	Non-inferiority one-sided p-value (+2 stool margin)	<0.001	<0.001	
		
Total loose or watery stools after enrolment (Per protocol analysis)	N	1431	1437	1410
	Mean + SD	10.8 ± 8.9	11.0 ± 9.3	10.6 ± 8.2
	Mean difference and upper bound of one-sided 98.75% CI	0.2 (0.9)	0.3 (1.1)	Ref.
Vomiting Related Outcomes				

Proportion of children who ever vomited over 14 days within 30 minutes of dosing	n/N%	206 / 1504 (13.7)	233 / 1498 (15.6)	289 / 1498 (19.3)
	Relative risk (97.5% CI)^2^	0.71 (0.59-0.86)	0.81 (0.67-0.96)	Ref.
	Superiority two-sided p-value^2^	<0.001	0.007	
Proportion of children who ever vomited over 14 days after 30 minutes of dosing)	n/N%	301 / 1496 (20.1)	333 / 1488 (22.4)	403 / 1490 (27.0)
	Relative risk (95% CI)	0.74 (0.65-0.85)	0.83(0.73-0.94)	Ref.

1 A post-hoc Bonferroni correction was applied to the primary efficacy outcomes account for tests of the two efficacy outcomes and two treatment arms; 98.75% confidence intervals are presented and statistical significance of P-values should be assessed at a P < 0.0125 (0.05 / 4 tests).

2 A post-hoc Bonferroni correction was applied to the primary safety outcome account for tests of the two treatment arms; 97.5% confidence intervals are presented and statistical significance of P-values should be assessed at a P < 0.025 (0.05 / 2 tests).

For the second efficacy outcome, ITT analyses showed (Table 2) that the mean (SD) number of loose or watery stools were similar across all three zinc arms, 10.8 (8.9) in the 5 mg group, 10.9 (9.2) in the 10 mg group, and 10.7 (8.7) in the standard 20 mg group. Compared with those assigned to 20 mg, children assigned to 10 mg of zinc had a mean difference of 0.3 (upper bound of one-sided 98.75% CI 1.1) stools while children assigned to 5 mg of zinc had a mean difference of 0.1 (upper bound of one-sided 98.75% CI 0.9) stools. In both cases, the upper bound of 98.75% CI was less than the pre-defined 2 stools non-inferiority margin. Results from per protocol analyses were similar to those from the ITT analyses.

The effect of lower doses of zinc on the risk of vomiting is presented in Table 2. Compared to children in the 20 mg group, children assigned 5 mg of zinc had a 29% lower risk of vomiting within 30 minutes of zinc administration during the 14 days of therapy (RR 0.71, 97.5% CI 0.59 to 0.86). Children assigned 10 mg of zinc had a 19% lower risk of vomiting within 30 minutes of zinc administration during the 14 days of therapy (RR 0.81, 97.5% CI 0.67 to 0.96). Similar effects were seen on vomiting > 30 minutes after zinc administration.

Results of subgroup analyses of primary outcomes are shown in Supplementary Tables C, D and E. The effect of lower doses of zinc appear to have a larger beneficial effect on vomiting in India than in Tanzania. Use of Rotavirus vaccine, which is co-linear with site, shows the same results. All other subgroup analyses results are unremarkable.

### Secondary outcomes

**Figure 2:**
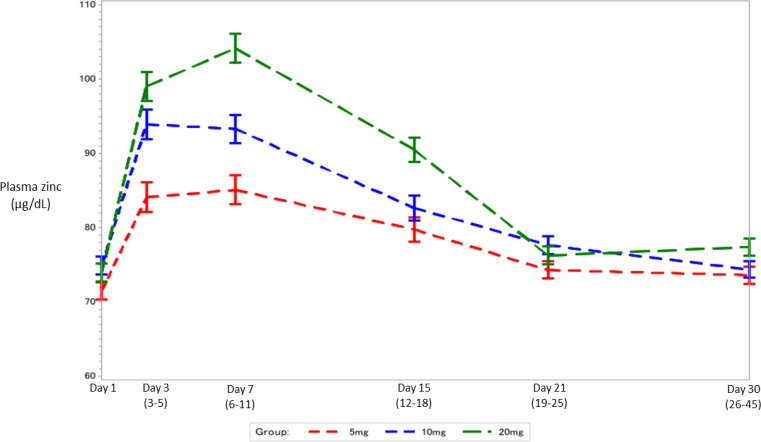
Modeled* mean plasma zinc concentration (µg/dL) and standard error at Day 1 (Baseline), Day 3, Day 8, Day 15, Day 21 and Day 30 by randomized treatment arm *Linear mixed-effects model estimates

Figure 2 shows mean and standard error of plasma zinc concentrations at Day 1(Baseline), Day 3, Day 8, Day 15, Day 21 and Day 30 time-bins by randomized treatment arm. Plasma zinc concentrations were similar at baseline but are lower in the 5 mg and 10 mg arms compared with 20 mg arm on days 3, 7 and 14 after dosing. These differences are no longer seen on day 21 and 30 (data presented in Supplementary F).

Secondary outcome results are shown in Table 3. The three study groups appear to have similar proportion of children experiencing diarrheas, fevers, or episodes of fast or difficult breathing at 30, 45 or 60 days follow up. Growth during the 60-day follow up period and anthropometric status as the end of follow up was also similar across study groups, except for a higher proportion of stunted children in 5 mg group. However, the observed difference in stunting between groups was the same as that at baseline. Adherence to the intervention was very high and similar across the three groups. Of the expected 14 pills, 13.5 (SD 2.1), 13.7 (SD 1.7) and 13.6 (SD 1.8) were consumed by children in the 5 mg, 10 mg and 20 mg groups respectively. Over 80% of mothers in all three groups (82.9% in the 5 mg group, 80.8% in the 10 mg group, and 81.1 in the standard 20 mg group) reported favorable acceptance by their child.

**Table 3:** Effect of zinc supplementation dose on secondary outcomes.

Secondary Outcomes					
	5 mg (n=1504) Mean±SD or n(%)	Effect size (95% CI)	10 mg (n=1498) Mean±SD or n(%)	Effect size (95% CI)	20 mg (n=1498) Mean±SD or n(%)
Proportion of children with SAEs within 60 days	12 (0.8)	1.00 (0.46,2.14)	7 (0.5)	0.69 (0.30,1.61)	9 (0.6)
Proportion of children with diarrhoea continuing beyond 3 days (n=1481,1481,1480)	333 (22.5)	1.03 (0.90-1.18)	333 (22.5)	1.03 (0.90-1.18)	323 (21.8)
Mean number of tablets consumed during 14 day treatment	13.53±2.05	-0.05 (-0.19,0.09)	13.65±1.71	0.06 (-0.06,0.19)	13.58±1.83
Number of follow up visits with diarrhea in the 2week period before day 30,45 and 60 / Total number of follow up visits*	233/4319 (5.4)	0.87 (0.71,1.06)	258/4351 (5.9)	0.95 (0.79,1.15)	269/4331 (6.2)
Proportion of children who had diarrhea in the 2-week period before day 30 (n=1433,1449,1437)	104 (7.3)	0.90 (0.70,1.16)	104 (7.2)	0.89 (0.69,1.15)	116 (8.1)
Proportion of children who had diarrhea in the 2-week period before day45 (n=1434,1444,1447)	60 (4.2)	0.80 (0.57,1.11)	77 (5.3)	1.02 (0.75,1.38)	76 (5.3)
Proportion of children who had diarrhea in the 2-week period before day 60 (n=1452,1458,1447)	69 (4.8)	0.89 (0.65,1.23)	77 (5.3)	0.99 (0.73,1.35)	77 (5.3)
Number of follow up visits with fever in the 2-week period before day 30,45 and 60 / Total number of follow up visits^1^	388/4319 (9.0)	0.99 (0.85,1.15)	397/4351 (9.1)	1.00 (0.86,1.16)	394/4331 (9.1)
Proportion of children who had fever in the 2-week period before day 30 (n=1433,1449,1437)	149 (10.4)	1.12 (0.90,1.40)	139 (9.6)	1.04 (0.83,1.30)	133 (9.3)
Proportion of children who had fever in the 2week period before day 45 (n=1434,1444,1447)	113 (7.9)	0.85 (0.67,1.08)	124 (8.6)	0.93 (0.73,1.17)	134 (9.3)
Proportion of children who had fever in the 2week period before day 60 (n=1452,1458,1447)	126 (8.7)	0.99 (0.78,1.25)	134 (9.2)	1.05 (0.83,1.32)	127 (8.8)
Number of follow up visits with fast or difficult breathing in the 2-week period before day 30,45 and 60 / Total number of follow up visits*	26/4318 (0.60)	1.19 (0.65,2.18)	34/4351 (0.78)	1.54 (0.85,2.79)	22/4328 (0.51)
Proportion of children who had fast or difficult breathing in the 2-week period before day 30 (n=1432,1449,1436)	8 (0.6)	1.34 (0.47,3.84)	10 (0.7)	1.65 (0.60,4.53)	6 (0.4)
Proportion of children who had fast or difficult breathing in the 2-week period before day 45 (n=1434,1444,1445)	11 (0.8)	1.23 (0.51,2.96)	14 (1.0)	1.56 (0.68,3.59)	9 (0.6)
Proportion of children who had fast or difficult breathing in the 2-week period before day 60 (n=1452,1458,1447)	7 (0.5)	1.00 (0.35,2.83)	10 (0.7)	1.42 (0.54,3.71)	7 (0.5)
Proportion of mothers who report of ease in supplement administration (n=1447,1453,1452)	1187 (82.0)	1.01 (0.97,1.04)	1174 (80.8)	0.99 (0.96,1.03)	1182 (81.4)
Mean change in length (1356,1357,1357)	1.54±0.89	-0.04 (-0.11,0.03)	1.58±0.89	0.003 (-0.06,0.07)	1.58±0.90
Mean change in weight (1367,1370,1363)	0.51±0.42	0.03 (-0.002,0.06)	0.50±0.41	0.02 (-0.007,0.05)	0.48±0.41
Mean change in MUAC (1345,1348,1356)	0.33±0.45	0.03 (-0.007,0.06)	0.33±0.46	0.03 (-0.004,0.07)	0.30±0.46
Mean change in LAZ/HAZ (1355,1357,1357)	-0.11±0.34	-0.01 (-0.04,0.01)	-0.10±0.34	-0.005 (-0.03,0.02)	-0.10±0.33
Mean change in WAZ (1367,1370,1363)	0.10±0.35	0.02 (-0.007,0.05)	0.10±0.35	0.02 (-0.004,0.05)	0.08±0.35
Mean change in WLZ/WHZ (1354,1350,1360)	0.19±0.54	0.03 (-0.01,0.07)	0.18±0.55	0.03 (-0.01,0.07)	0.16±0.54
Mean change in MUAC Z score 1333,1330,1346)	0.21±0.40	0.02 (-0.008,0.05)	0.21±0.40	0.03 (-0.005,0.06)	0.18±0.41
Proportion stunted (1354,1356,1357)	405 (29.9)	1.16 (1.03,1.31)	386 (28.5)	1.11 (0.98,1.25)	350 (25.8)
Proportion wasted (1354,1350,1360)	86 (6.4)	0.85 (0.64.1.12)	102 (7.6)	1.01 (0.77,1.31)	102 (7.5)
Proportion underweight (1367,1370,1363)	284 (20.8)	1.08 (0.93,1.26)	273 (19.9)	1.04 (0.89,1.21)	262 (19.2)
Proportion of study dropouts	48 (3.2)	1.00 (0.67,1.48)	43 (2.9)	0.89 (0.60,1.34)	48 (3.2)
Proportion of mothers who report favorable child acceptability (n=1447,1453,1452)	1199 (82.9)	1.02 (0.99,1.06)	1174 (80.8)	1.00 (0.96,1.03)	1177 (81.1)
Proportion of mothers who are willing to recommendation for use for other children	1427 (98.3)	1.00 (0.99,1.01)	1426 (97.9)	0.99 (0.98,1.00)	1431 (98.4)
Proportion of mothers who report improvement in child's skin condition (n=1446,1453,1453)	199 (13.8)	1.05 (0.87,1.27)	200 (13.8)	1.05 (0.88,1.27)	190 (13.1)
Proportion of mothers who report improvement in child's appetite (n=1446,1453,1453)	609 (42.1)	1.04 (0.96,1.14)	606 (41.7)	1.03 (0.95,1.13)	587 (40.4)
Proportion of mothers who report improvement in child's activity level (n=1446,1453,1453)	571 (39.5)	1.00 (0.92,1.10)	580 (39.9)	1.02 (0.93,1.11)	571 (39.3)
Proportion of mothers who report improvement in child's mood (n=1446,1453,1453)	619 (42.8)	1.03 (0.95,1.13)	620 (42.7)	1.03 (0.95,1.12)	602 (41.4)
Proportion of mothers who report reduction in diarrhea severity (n=1446,1453,1453)	1300 (89.9)	1.00 (0.97,1.02)	1315 (90.5)	1.00 (0.98,1.03)	1309 (90.1)

1 Generalized estimating equations (GEE) with the log link, binomial distribution and exchangeable correlation matrix was used to estimate the relative risk and robust estimators of the variances were used to construct 95% confidence intervals.

## Discussion

In this large, multicenter clinical trial, lower doses of zinc (5 or 10 mg daily for 14 days) was non-inferior to standard dose zinc (20 mg) in terms of duration of diarrhea and mean stool number after enrollment in children with acute diarrhea. Both the 5 and 10 mg doses were superior to the standard 20 mg dose with respect to vomiting.

Vomiting is often a part of the acute diarrhea syndrome. The additional contribution of high dose zinc therapy is important, with meta-analyses of previous trials demonstrating a 50% higher risk of vomiting in zinc supplemented children^4,12^. Efforts to scale up wider use of zinc have highlighted the elevated risk of vomiting as a consideration in programmatic roll-out.^20^ Reduced vomiting may improve food intake and alleviate parents’ concerns about severity of illness. Our data indicate that adherence to therapy was very high in all groups, with no difference between the groups. It is noteworthy, however, that our study was an efficacy trial in which substantial efforts were made to achieve high compliance. Thus, these adherence findings might not be generalizable to program conditions and reduced vomiting could potentially improve adherence under those conditions.

An interesting finding of our trial was the potential effect modification noted by site; Indian children appeared to benefit more from lower zinc doses with respect to vomiting than did children in Tanzania. In addition to country of origin and likely numerous other unmeasured factors, children in the two sites differed by age and rotavirus vaccine coverage rates. Although we did not collect data on the etiology of diarrhea in our subjects, it is possible that Indian children were more likely to have rotavirus as a cause of their symptoms^22^. In countries where rotavirus vaccine has been implemented, norovirus and sapovirus are common etiologies of childhood diarrhea ^23^. Only limited data exist on the potential differential effect of supplemental zinc based on diarrhea etiology ^24^. Stratified analyses also suggested the possibility that children with stunting, who may be at higher risk of adverse effects of diarrheal diseases ^25,26^, experienced greater reduction in the risk of vomiting with the lower zinc doses compared to the standard 20 mg zinc regimen.

The physiologic basis for the effects of zinc supplementation for diarrheal diseases is not completely clear ^27^. Possible mechanisms include the correction of a nutrient deficiency, the improvement of immune function ^28^, and/or the inhibition of cAMP-mediated chloride secretion^29^. The dosages we studied in the trial (10 and 5 mg daily) still exceed the RDA for young children, and therefore it is plausible that they will still be able to work through these suggested mechanisms of action

Strengths of our trial include its randomized double-blind multicenter design with a large sample size and excellent rates of follow-up, its location in countries in south Asia and sub-Saharan Africa, and its recruitment of patients in outpatient facilities, where the majority of diarrhea is managed globally. Our study addresses an important knowledge gap presented by the empiric use of the high dose of 20 mg, which was recommended for global use without rigorous dose-finding trials.

Limitations include the lack of a placebo arm (which was unethical given the volume of data supporting the efficacy of zinc supplementation), and reliance on caretaker report for study outcomes (although these were verified with frequent subject contact and daily recording of outcome information in the diary card). In addition, the modest rate of participation of children with severe diarrheal disease likely affected our lower than anticipated rate of children whose episodes lasted >5 days, since this outcome was less frequent than we had hypothesized for sample size calculations. However, our trial demonstrated non-inferiority of lower doses of zinc for this outcome. Our patients, however, are representative of those who present with diarrhea to first level health facilities in low- and middle-income countries and are given ORS and zinc treatment.

Despite evidence supporting the efficacy of supplemental zinc in improving outcomes in diarrhea, and strong recommendations from policy makers, programmatic uptake of this component of diarrhea management has been slow to achieve high coverage levels ^1^. Evaluations of this limited coverage highlight the supply side problems of insufficient financial and human capital and a weak global supply chain^21^. A renewed public health push is required to solve these problems and maximize the benefits of this efficacious intervention to vulnerable children. Our findings may contribute to these programmatic efforts.

In summary, children with acute diarrhea receiving 5 or 10 mg per day of supplement zinc had similar diarrhea outcomes but less vomiting, compared with children who received the standard 20 mg dose.

## Data Availability

The datasets generated during and/or analyzed during the current study will be made available from the corresponding author on reasonable request.

## References

[1] Black R, Fontaine O, Lamberti L, et al. Drivers of the reduction in childhood diarrhea mortality 1980-2015 and interventions to eliminate preventable diarrhea deaths by 2030. Journal of global health 2019;9:020801. 31673345 10.7189/jogh.09.020801 PMC6815873

[2] WHO/UNICEF Clinical management of acute diarrhoea. Geneva: WHO; 2004 Report No.: WHO/FCH/CAH 04.7.

[3] Patel A, Mamtani M, Dibley MJ, Badhoniya N, Kulkarni H Therapeutic value of zinc supplementation in acute and persistent diarrhea: a systematic review. PLoS One 2010;5:e10386. 20442848 10.1371/journal.pone.0010386 PMC2860998

[4] Lukacik M, Thomas RL, Aranda JV A Meta-analysis of the Effects of Oral Zinc in the Treatment of Acute and Persistent Diarrhea. Pediatrics 2008;121:326-36. 18245424 10.1542/peds.2007-0921

[5] Brooks WA, Santosham M, Roy SK, et al. Efficacy of zinc in young infants with acute watery diarrhea. Am J Clin Nutr 2005;82:605-10. 16155274 10.1093/ajcn.82.3.605

[6] Bhatnagar S, Bahl R, Sharma PK, Kumar GT, Saxena SK, Bhan MK Zinc With Oral Rehydration Therapy Reduces Stool Output and Duration of Diarrhea in Hospitalized Children: A Randomized Controlled Trial. Journal of Pediatric Gastroenterology & Nutrition 2004;38:34-40. 14676592 10.1097/00005176-200401000-00010

[7] Strand TA, Chandyo RK, Bahl R, et al. Effectiveness and Efficacy of Zinc for the Treatment of Acute Diarrhea in Young Children. Pediatrics 2002;109:898-903. 11986453 10.1542/peds.109.5.898

[8] Zinc Investigators Collaborative Group Therapeutic effects of oral zinc in acute and persistent diarrhea in children in developing countries: pooled analysis of randomized controlled trials. Am J Clin Nutr 2000;72:1516-22. 11101480 10.1093/ajcn/72.6.1516

[9] Sazawal S, Black RE, Bhan MK, Bhandari N, Sinha A, Jalla S Zinc supplementation in young children with acute diarrhea in India. N Engl J Med 1995;333:839-44. 7651474 10.1056/NEJM199509283331304

[10] Standing Committee on the Scientific Evaluation of Dietary Reference Intakes - Food and Nutrition Board - Institute of Medicine Dietary Reference Intakes for Vitamin A, Vitamin K, Arsenic, Boron, Chromium, Copper, Iodine, Iron, Manganese, Molybdenum, Nickel, Silicon, Vanadium, and Zinc. Washington, DC: National Academy Press; 2002.

[11] Larson CP, Hoque A, Larson C, Khan A, Saha U Initiation of Zinc Treatment for Acute Childhood Diarrhoea and Risk for Vomiting orRegurgitation: A Randomized, Double-blind, Placebo-controlled Trial. J Health Popul Nutr 2005;23:311-9. 16599101

[12] Lazzerini M, Ronfani L Oral zinc for treating diarrhoea in children. Cochrane database of systematic reviews 2008:CD005436. 18646129 10.1002/14651858.CD005436.pub2

[13] Somji SS, Dhingra P, Dhingra U, et al. Effect of dose reduction of supplemental zinc for childhood diarrhoea: study protocol for a double-masked, randomised controlled trial in India and Tanzania. BMJ Paediatr Open 2019;3:e000460. 10.1136/bmjpo-2019-000460 PMC6542451 31206083

[14] International Zinc Nutrition Consultative Group Assessing population zinc status with serum zinc concentration. IZINCG technical brief no. 22012.

[15] Bahl R, Bhandari N, Saksena M, et al. Efficacy of zinc-fortified oral rehydration solution in 6- to 35-month-old children with acute diarrhea. J Pediatr 2002;141:677-82. 12410197 10.1067/mpd.2002.128543

[16] Mauri L, D'Agostino RB Sr. Challenges in the Design and Interpretation of Noninferiority Trials. N Engl J Med 2017;377:1357-67. 28976859 10.1056/NEJMra1510063

[17] Spiegelman D, Hertzmark E Easy SAS calculations for risk or prevalence ratios and differences. Am J Epidemiol 2005;162:199-200. 15987728 10.1093/aje/kwi188

[18] Pedroza C, Thanh Truong VT Performance of models for estimating absolute risk difference in multicenter trials with binary outcome. BMC Med Res Methodol 2016;16:113. 27576307 10.1186/s12874-016-0217-0 PMC5006411

[19] WHO Handbook: IMCI integrated management of childhood illness. Geneva, Switzerland: World Health Organization; 2005.

[20] Larson CP, Koehlmoos TP, Sack DA, Scaling Up of Zinc for Young Children Project T. Scaling up zinc treatment of childhood diarrhoea in Bangladesh: theoretical and practical considerations guiding the SUZY Project. Health Policy Plan 2012;27:102-14. 21343236 10.1093/heapol/czr015

[21] Gill CJ, Young M, Schroder K, et al. Bottlenecks, barriers, and solutions: results from multicountry consultations focused on reduction of childhood pneumonia and diarrhoea deaths. The Lancet 2013;381:1487-98. 10.1016/S0140-6736(13)60314-1 23582720

[22] Chandola TR, Taneja S, Goyal N, et al. Descriptive epidemiology of rotavirus infection in a community in North India. Epidemiol Infect 2013;141:2094-100. doi: 10.1017/S0950268812002762 Epub 2013 Jan 8. 23298643 PMC9151399

[23] Becker-Dreps S, Bucardo F, Vilchez S, et al. Etiology of childhood diarrhea after rotavirus vaccine introduction: a prospective, population-based study in Nicaragua. Pediatr Infect Dis J 2014;33:1156-63. 24879131 10.1097/INF.0000000000000427 PMC4216626

[24] Patel AB, Dibley MJ, Mamtani M, Badhoniya N, Kulkarni H Influence of zinc supplementation in acute diarrhea differs by the isolated organism. Int J Pediatr 2010;2010:671587. 20592753 10.1155/2010/671587 PMC2879540

[25] Olofin I, McDonald CM, Ezzati M, et al. Associations of suboptimal growth with all-cause and cause-specific mortality in children under five years: a pooled analysis of ten prospective studies. PLoS One 2013;8:e64636. 23734210 10.1371/journal.pone.0064636 PMC3667136

[26] Santosham M, Chandran A, Fitzwater S, Fischer-Walker C, Baqui AH, Black R Progress and barriers for the control of diarrhoeal disease. Lancet 2010;376:63-7. 20609988 10.1016/S0140-6736(10)60356-X

[27] Hoque KM, Binder HJ Zinc in the treatment of acute diarrhea: current status and assessment. Gastroenterology 2006;130:2201-5. 16762641 10.1053/j.gastro.2006.02.062

[28] Rahman MJ, Sarker P, Roy SK, et al. Effects of zinc supplementation as adjunct therapy on the systemic immune responses in shigellosis. Am J Clin Nutr 2005;81:495-502. 15699240 10.1093/ajcn.81.2.495

[29] Hoque KM, Rajendran VM, Binder HJ Zinc inhibits cAMP-stimulated Cl secretion via basolateral K-channel blockade in rat ileum. Am J Physiol Gastrointest Liver Physiol 2005;288:G956-63. 15618279 10.1152/ajpgi.00441.2004

