# Supplementary material for: Lower dose zinc for childhood diarrhea: a randomized, multicenter trial
Source: N Engl J Med. Author manuscript; Available in PMC 2020 Sep 24. (PMC7466932; doi:10.1056/NEJMoa1915905)
Supplement: Supplementary file 1 [file NEJM-2020-1915905-s1.pdf]

Appendix

Contents

List of Investigators 2

Methods Paper 3

Supplementary Figures 21

Supplementary Tables 24

References 43

List of Investigators

Usha Dhingra, M.C.A.[1]; Pratibha Dhingra, Ph.D.[1], Arup Dutta, M.B.A.[1], Prabhabati Devi, Ph.D.[1], Saikat Deb, Ph.D[1], Aishwarya Chauhan, Ph.D.[1], Jitendra Kumar, Ph.D.[1], Om Prakash Semwal, D.C.H.[1], Sunil Sazawal, Ph.D.[1], Karim Manji, M.B.B.S.[2], Sarah Somji, M.P.H.[2], Rodrick Kisenge, Ph.D.[2], Mohamed Bakari, M.Sc.[2], Said Aboud, Ph.D.[2], Enju Liu, Ph.D.[3], Christopher R. Sudfeld, Sc.D.[4], Christopher P. Duggan, M.D.[3,4], Rajiv Bahl, Ph.D.[5], Per Ashorn, M.D.[5], Jonathon Simon, D.Sc.[5].

[1] Center for Public Health Kinetics, New Delhi, India

[2] Muhimbili University of Health and Allied Sciences, Dar es Salaam, Tanzania

[3] Boston Children's Hospital, Boston, MA, USA

[4] Harvard TH Chan School of Public Health, Boston, MA, USA

[5] World Health Organization, Maternal, Newborn, Child, Adolescent Health and Ageing Department, Geneva, Switzerland

Methods Paper

Somji SS, *et al*. *BMJ Paediatrics Open* 2019;3:e000460. doi:10.1136/bmjpo-2019-000460 1

Effect of dose reduction of supplemental zinc for childhood diarrhoea: study protocol for a doublemasked, randomised controlled trial in India and Tanzania

Sarah S Somji,[1] Pratibha Dhingra,[2] Usha Dhingra,[2] Arup Dutta,[2] Prabhabati Devi,[2]
Jitendra Kumar,[2] Saikat Deb,[2] Om Prakash Semwal,[2] Sunil Sazawal,[2] Karim Manji,[1]
Rodrick Kisenge,[1] Mohamed Bakari,[1] Said Aboud,[1] Enju Liu,[3]
Christopher Sudfeld,[4] Christopher P Duggan,[5,6] Per Ashorn,[7] Rajiv Bahl,[8]
Jonathon L Simon [8]

To cite: Somji SS, Dhingra P, Dhingra U, *et al*. Effect of dose reduction of supplemental zinc for childhood diarrhoea: study protocol for a double masked, randomised controlled trial in India and Tanzania.

BMJ Paediatrics Open
2019;3:e000460. doi:10.1136/bmjpo-2019-000460

►► Additional material is published online only. To view, please visit the journal online (http:// dx. doi. org/ 10. 1136/ bmjpo- 2019- 000460).

SSS and PD are joint first authors

Received 13 February 2019
Revised 28 February 2019
Accepted 7 March 2019
For numbered affiliations see end of article.

Correspondence to
Dr Jonathon L Simon; simonjo@
who. int

© Author(s) (or their employer(s)) 2019.
Re-use
permitted under CC BY-NC. No commercial re-use.
See rights and permissions.
Published by BMJ.

Abstract

Background Diarrhoea-associated mortality and morbidity are highest in infants and young children in low-income and middle-income countries (LMICs). Zinc supplementation during acute diarrhoea has been shown to reduce the duration of illness and the risk of persistent diarrhoea. However, vomiting with zinc supplementation is a common side effect that may interfere with compliance and programmatic scale-up, and may be related to the dose prescribed.

Methods/design The Zinc Therapeutic Dose Trial
(ZTDT) is a two-centre (Tanzania and India), three-arm randomised, double-blind controlled non-inferiority trial.
Children 6–59 months of age with acute diarrhoea are eligible to participate. Enrolled children (1500 per arm; 4500 total) will be randomly allocated to receive 5, 10 or 20 mg of zinc sulfate daily for 14 days and will be followed up for 60 days after enrolment. All children will receive WHO/Unicef Integrated Management of Childhood Illness standard of care (oral or intravenous rehydration and zinc as indicated and feeding advice). The primary efficacy outcomes of the trial are the percentage of subjects with diarrhoea duration >5days, the mean total number of loose or watery stools after enrolment and the proportion of children vomiting within 30 min of zinc administration.

Discussion The ZTDT trial will determine the optimal dose of therapeutic zinc supplements for treatment of acute diarrhoea in children aged 6–59 months in two LMICs. The results of the trial are likely to be generalisable to childhood acute diarrhoea in similar resource-limited settings and may influence global policy about zinc supplementation dosage during acute diarrhoea.

Trial registration number NCT03078842.

Trial status Enrolment began in January 2017 and follow-up is estimated to be completed by April 2019. As of 1 February 2019, 742 children are still contributing data to the ZTDT study.

What is already known on this topic?

- Therapeutic zinc supplementation has been recommended by WHO/Unicef for standard diarrhoea case management since 2004.
- Research evidence had shown zinc reduced the duration and severity of the acute diarrhoea, and lowered the risk of persistent diarrhoea.
- However, many trials noted that current dosage (20 mg/day for children aged >6 months) is associated with vomiting and possibly poor compliance.

What this study hopes to add?

- This study evaluates whether lower doses of zinc (10 mg/5 mg) are non-inferior to the standard dose in efficacy but superior for vomiting.
- If vomiting is less frequent and efficacy is maintained with low-dose zinc, caregiver acceptability may improve, leading to greater utilisation and better health outcomes.

Background

Zinc is a vital micronutrient essential for protein synthesis, cell growth and differentiation, immune function, and intestinal transport of water and electrolytes.[1] Zinc is also important for normal growth and development of children both with and without diarrhoea.[2] Zinc deficiency is associated with an increased risk of gastrointestinal infections, altered structure and function of the gastrointestinal tract and impaired immune function. [3–5] Dietary deficiency of zinc is especially common in low-income countries because of a low dietary intake of zinc-rich foods (including foods of animal origin) or low bioavailability due to the presence of phytates often found in cereal-based diets.[1]

The benefits of zinc supplementation in the management of diarrhoea have been observed in several studies. Patel *et al*[6] noted in a systematic review and meta-analysis an approximate 20% reduction in the mean duration of acute diarrhoea with zinc supplementation. Lazzerini and Wanzira,[7] summarising data from 2581 children from 9 trials, noted that children older than 6 months receiving zinc supplements had a shorter diarrhoea duration by about half a day (mean difference −11.46 hours, 95% CI −19.72 to −3.19). A reduction in diarrhoea that continues up to day 7 (risk ratio 0.73, 95% CI 0.61 and 0.88) was also noted in this study. Moreover, the study indicated that zinc supplements in children with persistent diarrhoea (defined as diarrhoea for >14 days) also may shorten average diarrhoea duration by 16 hours (mean difference −15.84 hours, 95% CI −25.43 to −6.24). As a result of these and other trials, since 2005 WHO/Unicef have recommended routine zinc supplementation (10 mg daily for infants below 6 months of age; 20 mg daily for children older than 6 months) for 10–14 days.[8]

These doses, however, were not chosen after dose ranging studies, and substantially exceed the recommended dietary allowances (RDA) for zinc in infancy and early childhood (2–5 mg/day).[9] High doses of zinc can cause epigastric pain and lethargy, and its metallic taste can also induce vomiting. Vomiting with zinc supplements is a common side effect noted in many published trials. A meta-analysis by Lukacik *et al* [3] summarised the findings for acute and persistent diarrhoea from a total of 22 zinc intervention trials, using a range of zinc dosing (5–45 mg/day). In 11 acute diarrhoea trials (n=4438), the proportion of participants who vomited after the initial dose was significantly higher with zinc use than with placebo (12.7% vs 7.6%; relative risk [RR]: 1.55; 95% CI: 1.30 to 1.84; p≤0.001). According to the review by Lazzerini and Wanzira,[7] the risk of vomiting increased with zinc supplementation in both children older than age 6 months (risk ratio 1.57, 95% CI 1.32 to 1.86; 2605 children, 6 trials) as well as children younger than 6 months of age (risk ratio 1.54, 95% CI 1.05 to 2.24; 1334 children, 2 trials).
Other studies have also reported that therapeutic zinc supplementation during diarrhoea causes higher rates of vomiting.[10–12]

Thus, although substantial evidence supports the efficacy of zinc supplementation during episodes of diarrhoea, vomiting is significantly more common among supplemented children. Lower doses of zinc, provided they are equally effective, might have the advantage of reducing side effects, increasing compliance and programmatic roll-out. If the zinc dose for diarrhoea treatment and zinc deficiency can be uniform, then the national essential drug lists can be simplified. Therefore, there exists an important rationale to identify the dose of zinc supplementation associated with both efficacy and an improved safety profile. We present the protocol for a two-centre, randomised, double-blind controlled trial of three doses of zinc conducted in Tanzania and India. The overall goals of the study are to evaluate the non-inferiority, safety and acceptability of the two lower zinc doses (5 and 10 mg/day) versus standard zinc dose (20 mg/day).

Methods

Study design

The Zinc Therapeutic Dose Trial (ZTDT) is an individually randomised, parallel-group, double-blind, controlled trial of three doses of supplemental zinc conducted among 4500 children aged 6–59 months in India and Tanzania (ClinicalTrials. gov identifier NCT03078842).

The trial protocol was developed by collaborators at WHO, Department of Maternal Newborn, Child and Adolescent Health (Geneva, Switzerland) together with teams in New Delhi, India (Centre for Public Health Kinetics), Dar es Salaam, Tanzania (Muhimbili University of Health and Allied Sciences) and Boston, USA (Boston Children's Hospital and Harvard TH Chan School of Public Health). The ZTDT protocol diagram is presented in online supplementary additional file 1. The schedule of trial enrolment, interventions and assessments is presented in online supplementary additional file 2. The first participant was enrolled in the trial in January 2017 in India and March 2017 in Tanzania and follow-up data collection will continue through approximately April 2019. This paper follows the Standard Protocol Items: Recommendations for Interventional trials (SPIRIT) checklist (see online supplementary additional file 3).

The study population will include 4500 children aged 6–59 months with diarrhoea of <72 hours duration. Enrolled children will be followed up for 60 days after randomisation. The participant will be followed up closely for the first 15 days to record postintervention morbidity outcomes and to ensure compliance with study regimen. Study participants will be assessed again at 30, 45 and 60 days post-enrolment to estimate impact on long-term morbidity outcomes.

Patient and public involvement

This research was done without patient involvement. Patients were not invited to comment on the study design and were not consulted to develop patient relevant outcomes or interpret the results. Patients were not invited to contribute to the writing or editing of this document for readability or accuracy.

Primary and secondary objectives

he overall aim of ZTDT is to identify the optimal dose of zinc in children under 5 years with acute diarrhoea.

The primary aims of the study are: (1) to assess whether lower doses of zinc (5 or 10 mg/day) are at least as effective as the standard dose of zinc (20 mg/day) in children aged 6–59 months, in terms of burden of illness (percentage of subjects with diarrhoea lasting >5 days; mean number of loose or watery stools during follow-up) and (2) to evaluate the improved effectiveness by reduction in the risk of vomiting in 30 min after receipt of the supplement in low-dose supplemental zinc (5 or 10 mg/day) in comparison to standard dose of zinc (20 mg/day).

Secondary aims of the study are: (1) to explore whether lower doses of zinc have any impact on post-acute illness morbidity outcomes assessed at 30, 45 and 60 days, (2) to explore whether duration of treatment, estimated by mother's reports and collection of used zinc blister packs, has any impact on the primary outcomes, (3) to determine the acceptability of low-dose supplemental zinc compared with standard zinc dose based on an acceptability scale and (4) to determine whether the different doses of zinc are associated with differential plasma zinc levels at days 3, 7, 15, 21 and 30 after randomisation.

Participants/interventions and outcomes

Study setting

The study recruitment sites are in South East Asia (SEA) and sub-Saharan Africa (SSA). In SEA, the study recruitment sites are located in Sangam Vihar, a resettlement colony on outskirts of South Delhi and Harsh Vihar, a semi-urban locality in North East District, Delhi, India. In SSA, recruitment sites are at health centres in Temeke District Hospital, Mbagala Round Table and Mbagala Rangi Tatu, Dar es Salaam, Tanzania. Both the sites will be recruiting from outpatient health facilities.

Eligibility

Study participants will be eligible for enrolment in the study if they meet the following inclusion criteria: 1) male and female children between 6 and 59 completed months of age, 2) acute diarrhoea duration of <72 hours at the time of screening (defined as three or more loose or watery stools) or dysentery (defined as visible blood in the stool), 3) likely to stay within the study area for the next 2 months and 4) written informed consent is obtained from caretaker.

Children will be excluded if they: (1) have severe acute malnutrition (weight for length/height z-score of <−3 or oedema), (2) have severe dehydration that cannot be corrected within 4–6 hours, (3) have signs of severe pneumonia (characterised by presence of either chest in-drawing and either of the following: convulsions or unconscious or vomiting everything,13 sepsis, rapid diagnostic test (RDT)-confirmed malaria or other severe illness, (4) have previously or currently been enrolled in the study, (5) are currently enrolled in another trial, (6) are not intending to remain in study area for the duration of the study, (7) have a caregiver who declines participation in the study, (8) have other child currently enrolled in the study in the same household and (9) have used zinc supplements during the 3 days preceding study enrolment.

Sample size

We will enrol 4500 children (1500 per study arm) in the trial. Table 1 presents sample size assumptions and calculations for each of the three primary outcomes. The total sample size for the trial was selected to have at least 90% power for each of the primary outcomes. The primary outcomes (mean number of loose or watery stools post-randomisation and percentage of children with diarrhoea for >5 days) are both based on the non-inferiority hypotheses, while the primary outcome of vomiting post-dose assumes a superiority hypothesis. We assumed 1:1:1 randomisation between the three treatment arms,
a nominal type I error rate (α) of 0.05% and 5% loss to follow-up rate.

Outcome	Value in standard dose (20 mg/day) group	Expected value in low-dose groups	Non-inferiority margin	Power, significance level	Sample size per arm (with ~5% attrition)
Duration of diarrhoea, mean (SD)	3.0 (2.7) days	3.0 (2.7) days Lower dose equally effective	0.3 days	90%, 5%	1460
Percentage of children with diarrhoea >5 days	16%	16% Lower dose equally effective	±4%	90%, 5%	1500
Total number of loose or watery stools during follow-up, mean (SD)	10 (9) stools	10 (9) stools Lower dose equally effective	±2 stools	90%, 5%	1460
Percentage of children with vomiting during follow-up	20%	15% Lower dose safer	Not applicable because of superiority hypothesis for this outcome	90%, 5%	1340

Table 1 Sample size calculations for the primary trial outcomes

Interventions

Children are randomised to receive one of the three zinc sulfate regimens (each taken once daily for 14 days): (1) 5 mg; (2) 10 mg or (3) 20 mg. The zinc tablet (all three regimens) ingredients include aerosil,
maize starch, ethylvanillin (a vanilla flavour), aspartame, magnesium stearate vegetal and vivapur. The three study tablets have identical appearance, taste and smell. The supplement is a dispersible tablet that dissolves in water or breast milk in about 20 s. The caretakers are instructed to dissolve the tablet in 5–10 mL of water or breast milk immediately before administration. Children receive zinc supplementation for 14 days. The first dose of study drug is given at the health facility in which the child is enrolled.

All three doses of the regimen are packaged identically; 10 tablets in 1 blister, 2 blisters in 1 pack (extra doses are provided for redosing in case of vomiting). Each pack is prelabelled with participant ID. Study regimen is manufactured by Laboratoires Pharmaceutiques Rodael, France and shipped to WHO for randomization and labelling, before shipment to recruitment sites (as described in 'Randomisation, allocation, concealment and masking' section).

Adherence assessment

Adherence to the study regimen is optimised by close follow-up: either in person (clinic/home visits) or telephone contact on days 3, 5, 7, 10 and 15. Adherence to the regimen is measured by counting the numbers of unused tablets during each follow-up visit. Study workers/ research assistants physically count the tablets during home or clinic visits. We give the mother/caregiver a small booklet to record every successful administration as well as any failed attempts to administer the intervention. Study worker reviews the diary card on each visit and collects filled diary card on day 15 visit.

Standard of care

All enrolled children receive standard of care for diarrhoea management per WHO/Unicef, Tanzanian and Indian Ministry of Health guidelines. This includes standardised assessment of hydration status, rehydration and maintenance fluids with intravenous or oral rehydration solutions (ORS) as indicated, recommendation for increased fluid intake and continued feeding at home and instructions on when to seek follow-up care. Children with known HIV receive care and treatment appropriate for the child and referral to the care and treatment centre nearest to the child's home.

Randomisation, allocation, concealment and masking

Trial arm allocation was performed according to a computer-generated randomisation list (generated off-site by a non-study statistician) of 4500 individuals, stratified by country and child age (>6 to <24 months vs >24 to <60 months). A statistician will hold the randomization list codes until completion of primary analyses or until requested by the Data and Safety Monitoring Board (DSMB). Within each stratum, subjects are randomly assigned equally in permuted blocks of variable size to receive one of the three doses of zinc. The purpose of the stratification is to have equal distribution of younger and older children among all three treatment groups. An independent pharmacy team prepared the zinc regimen in Geneva by labelling blister packs with participant IDs based on the randomisation list. Labels were printed at WHO, Geneva and enrolled children were assigned sequential serial numbers. These prelabelled regimens were shipped to the sites in Tanzania and India. The packing and physical appearance of the three study regimes are identical. In addition, the three doses of the regimen are similar in taste, appearance and smell. As a result, the research teams, study staff as well as caregivers are fully blinded to treatment arm. Zinc formulations are serially numbered according to the randomisation codes. The code is kept in a password-protected spreadsheet file by WHO personnel and is not available to the investigators until the end of the study. This randomisation list was converted into unique sequential serial numbers for each enrolled child at each site. This code will only be broken if the DSMB requests this information for a participant (should they suspect a relationship of a suspected severe adverse event [SAE] to the study drug) or for a study arm (in their interim analyses).

Data collection, management and analysis

Screening and randomisation

Both sites will be recruiting from community clinics. Surveillance systems are set up at clinics to identify all children presenting with diarrhoea. Trained study staff stationed at each health facility are responsible for identification of children who are potentially eligible. The study team is trained in good clinical practice as well as confidentiality and research ethics.
Written informed consent is sought from the caretaker of children meeting the eligibility criteria. A child can only be enrolled once into the study. The informed consent process includes telling the caretaker that they are free to withdraw from the study at any time, and that participation is entirely voluntary.

Following consent, each participant will be assigned a unique subject identification number. A card detailing the subject ID and the health personnel responsible for enrolment will be provided to the caregiver of enrolled children. The patient locator form/subject master log will be the only link between the subject ID and the participant's name. This information will be stored together with the consent in a locked file cabinet and an online database accessible to only the Project Manager and required study staff for the purpose of patient tracking.

A baseline assessment, including a detailed physical examination (record of vital signs, axillary temperature, respiratory rate, pulse rate, hydration and nutritional status), will be performed by a research physician at the time of screening for enrolment. The child is evaluated for any Integrated Management of Childhood Illness general danger signs (unable/able to drink or breast feed, convulsions, vomiting, unconscious, stiff neck) and evidence of dehydration or pneumonia, as well as information on recent medical history (duration of diarrhoea, bloody stool/dysentery, recent fever). Vital signs temperature/heart rate/respiratory rate) in dehydrated child are recorded after the child is rehydrated and stabilised and remaining clinical assessment/screening is completed thereafter. Anthropometry measurements will be taken after rehydration and stabilisation as required, before completing the screening for enrolling the participant. Weight will be measured using an electronic scale with a sensitivity of ±10 g by two independent observers: length (for children aged <24 months) or height (for children aged 24 months or older), which will be measured using a length board within 0.1 cm. Mid-upper arm circumference (MUAC) will be measured using non-stretchable tape to the nearest 0.1 cm. All anthropometric measures will be recorded in duplicate (India) and triplicate (Tanzania). Once these measurements and other eligibility criteria are met, the subject will get enrolled.

Baseline sociodemographic information and vaccination history

Parents of enrolled subjects will be interviewed at study clinic or at home for the baseline sociodemographic characteristics viz. parent's literacy level and occupation, type of house, possession of assets and possible exposures (recent antibiotic use, travel history, water use and filtration and sanitation), breast feeding and vaccination history and information is recorded in the case record forms.

Follow-up visits

Scheduled follow-up visits begin from the third day of the enrolment and are generated daily via an automated visit generation module of the data management software. All children enrolled at the study clinic are to be followed up in the clinic or at home by a trained field worker on day 3, 5, 7, 10, 15, 30, 45 and 60 (online supplementary additional files 1 and 2). In India, study staff will visit the household of enrolled children for all follow-up visitations. If the child is not available at home, a phone call will be made to follow-up the child. In Tanzania, a participant will be followed up at home or clinic on days 3, 7, 15, 30 and 60 and by phone on days 5, 10 and 45. Children still having diarrhoea after day 15 are treated according to standard management guidelines.[8] At each visitation during active follow-up period, a study worker asks the mother or other caregiver about the following and records the responses in a predesigned form/data entry module:

1. Ongoing symptoms of diarrhoeal illness, including the number and consistency of stools.
2. Vomiting episodes including any episode within 30 min following zinc administration.
3. Adherence with zinc study regimen ascertained via mothers' recall and/or reported use of zinc tablets by the child in the Diarrhoea Diary Card and crosschecked by determining the number of unused tablets.
4. Any adverse events, including hospital admissions and deaths of study participants.
5. Criteria for returning to clinic (eg, lethargy, poor oral intake, dehydration).
6. Any other intercurrent illness.
7. Reported use of antibiotics and intravenous/other fluids.

At days 30, 45 and 60 post-enrolment, the study participants are assessed either at the study clinic, via telephone or with a home visit for presence of any illness, diarrhoea, fever, cough or fast or difficult breathing within last 24 hours and any time in past 2 weeks. On the day 60 study completion clinic visit, in addition to morbidity assessment, anthropometric measurements will be taken for each participant using the same procedure as mentioned in the baseline assessment.

Children not available on the visitation day will be visited the next day; if they are still not available, they are followed-up until found or deemed lost to follow-up. Caregivers are asked to contact the study physicians if they feel that their children are sick between the visits. Children sick on the day of visitation will be referred to the study clinic for a detailed morbidity assessment as well as treatment by the study physician. During each visit, a record of all the medicines and intravenous fluids given, information on number and consistency of stools and data on adherence is recorded in the predesigned forms. Close monitoring will be accomplished through repeated follow-ups during the 14-day regimen period. Every effort will be made to track and successfully follow-up all randomised children.

Study withdrawal or discontinuation

The participants have the right to withdraw from the study at any time. The subjects will be considered withdrawn from the trial in case of death or lost to follow-up. However, every effort will be made to track and successfully follow-up all enrolled children. The information on withdrawn or discontinued participants will be recorded through a withdrawal slip by field worker and clinic supervisor.

Blood sampling and laboratory investigations

To allow the measurement of the potential differential impact of the varying zinc doses on plasma zinc levels during the follow-up period, each study participant will provide two blood samples. One-third of the study participants will have blood draws at days 1 and 15, one-third at days 3 and 21 and the remaining one-third on days 7 and 30 post-enrolment for assessment of plasma zinc levels.

Blood drawing will follow the protocols of the International Zinc Nutrition Consultative Group (IZINCG). These include use of powder-free gloves, zinc-free syringes and butterfly, needles and trace element-free blood tubes.[14] Plasma is separated at 15 min after blood collection and aliquots are transferred into trace element-free Eppendorf plastic tubes for temporary storage at −20°C and then shipped to central lab for final storage at −80°C in India. In Tanzania, plasma samples are stored in site refrigerators at 2°C–8°C and then shipped to central laboratory storage at −80°C by the end of the day. Plasma zinc concentrations will be measured by atomic absorption spectroscopy (AAS-400 PerkinElmer, USA).

Quality assurance

The persons with at least a secondary school degree will be recruited for data collection team. The following quality assurance checks will be done regularly to ensure highest level of data integrity and quality:

- ►► An extensive 1–3 weeks training session for data collectors to ensure the interdata collector reliability is above 95%.
- ►► Pilot field data testing is monitored closely by the research scientist(s) responsible for quality assurance.
- ►► Real-time electronic data capture will ensure data validation such as range, logical checks and data integrity.

►► Interim aggregate data analysis will be carried out periodically for data pattern, heaping, missing values, etc. Based on the feedback, effective measures will be taken to eliminate/minimise the errors.

For overall visit supervision, quality control (QC) supervisors will perform quality checks by visiting at least 10% of the households, re-interviewing 5% respondents and cross-checking these data with concerned field worker. QC supervisors are primarily responsible for overall field-related project activities (data quality and integration at all levels, resolving community issues), and training. Computer randomised list of enrolled children will be used by QC team for random household visits.

The QC team works in close coordination with the investigative team and provides regular feedback on quality issues. Quarterly meetings between QC team and senior investigative team are scheduled to discuss overall quality issues and related corrective measures to be undertaken.

Data management

Data are collected in real-time using either tablets or laptop computers. Each site uses an in-house designed data management software with user friendly graphical user interface for data collection. Generic case report forms and data dictionary are used to maintain data consistency across sites. In-built range, logical and skipping pattern will ensure data quality and integrity. The database is password protected with restricted access. Each user is assigned a unique user identifier to access the software and an audit trial is maintained for database operations. The centralised server located at each site synchronises the data on daily basis. Data are shared monthly with WHO data quality monitoring team
who is responsible for data quality checks and provide feedback to sites. They generate query forms which are returned to the study sites for verification.

Data security and storage

A backup of the data is kept in the server at WHO protected by a specific password and accessible to only authorised users. An additional backup of the data is kept in a password-protected external hard drive at a site away from WHO at the recruiting site central offices.

Record retention and archival

All the study documents including participant's source data and documents will be archived by the study sites after the completion of the study, till the time WHO informs in writing to the study sites that they no longer need to maintain the study documents.

Outcome measures

The primary efficacy outcomes are 1) the percentage of enrolled children who have duration of diarrhoea of >5 days, 2) the mean number of loose or watery stools after enrolment and 3) the percentage of children vomiting within 30 min of administration of the zinc tablet.

Secondary outcomes of the trial include: i) proportion of children experiencing SAEs (life-threatening or hospitalisation); ii) proportion with intermediate duration of diarrhoea (diarrhoea continuing beyond 3 days); iii) proportion of guardians with positive attitude towards treatment; iv) treatment adherence; v) mean serum plasma zinc concentration at days 1, 3, 7, 15, 21 and 30; vi) illness symptoms between day 15 and 60 after the treatment 2-week period prevalence and number of days with diarrhoea, fever or

respiratory symptoms; vii) 60-day change in height/length, weight, MUAC, height/length-for-age z-score, weight-for-length/height z-score, weight-for-age z-score, MUAC z-score, stunting, wasting, underweight; viii) adherence to regimen; ix) study dropouts; x) maternal report of ease in supplement administration; xi) maternal report of child acceptability; xii) maternal recommendation of use for other children; xiii) maternal report of change in child skin condition, child appetite, activity levels, mood and diarrhoea severity.

Statistical analysis

All primary analyses of non-inferiority tests will be conducted on the principle of intention-to-treat (ITT).

All non-inferiority margins will be defined a priori. We will also conduct sensitivity analyses using a per-protocol (PP) approach.15 The analysis population for the ITT approach will include all randomised patients with assessable data, regardless of whether the participant is known to have received a full 14-day dose of zinc or not. The analysis population for the PP approach will include study participants who are documented to have taken zinc supplementation for the first 5 days after randomization regardless if they vomited the dose. The PP definition was selected due to the primary outcome being diarrhoea duration >5 days. Descriptive statistics will be used to summarise background demographic and baseline clinical characteristics by trial arm.

Analysis of primary outcomes

Primary outcome 1

Number (%) of subjects with diarrhoea episodes >5 days. The last day of diarrhoea will be defined as the day in which there is passage of the last liquid or semi-liquid stool prior to two diarrhoea-free days (days when less than three loose stools/day). The duration of diarrhoea will be defined as the number of days between randomisation (not the reported day of onset of diarrhoea symptoms before randomisation) and the last day with diarrhoea. Children who are lost to follow-up, die or withdraw before 5 days will be excluded from this analysis. For the primary analysis, we have set the threshold for non-inferiority of lower doses of zinc (10 or 5 mg/day) as compared with the standard 20 mg/day at <4% in absolute risk (risk difference) compared with the standard dose in the proportion of subjects with prolonged diarrhoea. The proportions of participants with a prolonged diarrhoea episode (defined as >5 days) will be calculated in all treatment arms, and the risk differences (and their 95% CIs) between the lower doses (5 and 10 mg/day) and the standard dose (20 mg) will be estimated. A Cochran-Mantel-Haenszel approach will be used to take stratifying randomisation factors, for example, country and age groups, into account. If the upper limit of the 95% CI for the risk difference is <4%, we will conclude non-inferiority for one or both lower dose arms. We will also examine the RR of prolonged diarrhoea episodes in both lower dose arms compared with the standard dose arm using RR regression which accounts for factors included in the stratified randomisation. In secondary analyses, Cox proportional hazard models will be used to estimate HRs and their 95% CIs for recovery from diarrhoea in each of the lower dose arms compared with the standard dose arm. The time to recovery is defined as the duration of diarrhoea as noted above.

Primary outcome 2

Total number of loose or watery stools after enrolment. An analysis of covariance (ANCOVA) approach will be used to estimate the mean differences and their 95% CI in the total number of loose or watery stools after enrolment for each lower dose arm compared with the standard dose arm,

while accounting for the stratified randomisation design. The non-inferiority margin was set at two stools, therefore if the upper limit of the 95% CI is less than two stools, we will reject the null hypothesis that the lower dose zinc is inferior to the standard dose in terms of reducing the total number of loose or watery stools.

Primary outcome 3

Vomiting within 30 min after administration of the trial regimen. We hypothesise that the lower dose of zinc will be associated with a reduced risk of vomiting and therefore a superiority test will be used. Vomiting within 30 min of each daily zinc dose is repeatedly measured during the 14-day administration period. Generalised estimating equations (GEE) with the log link, binomial variance and exchangeable correlation matrix will be used to assess the RR of vomiting among the three treatment arms. The exchangeable working covariance matrix and robust estimators of the variances will be used to construct CIs. The robust estimators are consistent estimators of the variances even if the working correlation matrix is misspecified. Stratified randomisation factors will be considered in the model. A secondary analysis concerning severe vomiting (defined as vomiting requiring hospitalization or vomiting requiring intravenous fluids) will also be performed in similar fashions. We will also examine the proportion of children ever vomiting between the treatment arms and examine whether the RR of vomiting differs by follow-up day.

Analysis of change in plasma zinc and other secondary outcomes

Due to the sampling design of participants having paired blood sampled on three schedules (days 1 and 15; days 3 and 21; days 7 and 30), we will assess the effect of the randomised zinc regimen at both the population level and individual level. For the analysis of population-level zinc, we will use a mixed model approach to compare patterns of plasma zinc concentrations over time between the randomised treatment arms. The main exposure variables in the model will include treatment arm, study day and an interaction term between treatment arm and study day. In case there is not a linear relationship between study day and plasma zinc concentrations, restricted cubic splines will be used to examine the non-linear relationship between these two variables. The interaction term between zinc dose and study day in the model measures whether the trajectory of plasma zinc differs by zinc supplementation over the full period. If the overall test for this interaction term is statistically significant, group means at each assessment day will then be compared using least-square means with the Tukey-Kramer adjustment. To be consistent with our stratified randomisation study design, stratification factors will be included in the model.

For all other secondary outcomes (see clinicaltrial.gov NCT03078842), we will use approaches similar to the primary analyses, including an ANCOVA approach for non-repeated continuous outcomes, RR regression for non-repeated binomial outcomes and GEE or mixed effect models for repeated longitudinal outcomes.

Sensitivity analyses and modification of treatment effect

Although randomisation is designed such that, on average, treatment groups will be balanced with respect to all baseline prognostic factors, this may not be true in all cases. As a result, we will re-assess treatment effects as observed in the original analysis after adjusting for factors that are potentially imbalanced across randomised arms. If imbalance occurs, we will compare and present the results for both unadjusted and adjusted analyses. In addition, the study was not designed to detect effect modification of the treatment effect among subgroups of children. As a result, we acknowledge that we are likely underpowered to detect differences in the magnitude of a treatment effect among subgroups unless there is strong effect modification. The potential baseline effect modifiers we plan to explore include: study site (India/Tanzania), child age

(<12 vs >12 months), child sex (male vs female), prior receipt of rotavirus vaccine (yes vs no), maternal HIV status (yes vs no), dysentery at enrolment (yes vs no), baseline dehydration status (yes vs no), baseline axillary temperature (<38°C vs >38°C), baseline respiratory rate, recent antibiotic use (yes vs no), intercurrent illness (cough, difficulty breathing or earache), current breastfeeding status (yes vs no) and prior breastfeeding status (yes vs no). Additional variables that we will test for effect modification will include child anthropometric measures (length/height for age, weight for age and weight for length/height z-scores and MUAC), family socioeconomic status (<median wealth index, >median wealth index), maternal education, household water quality (improved vs unimproved), household sanitation quality (improved vs unimproved), baseline plasma zinc levels (<median, >median) and trial regimen adherence (<median, >median). Factor analysis will be used to derive wealth index from family possession variables. WHO/Unicef Joint Monitoring Programme definitions will be used to clarify improved versus unimproved for the household water quality and sanitation.[16]

Monitoring

Data and Safety Monitoring Board

A Data Safety and Monitoring Board (DSMB) has been established to monitor SAEs and to review the statistical analysis plan and associated stopping rules for benefit, futility or harm determined using O'Brien-Fleming stopping boundaries. The DSMB includes five members with expertise in clinical trials, statistics, child mortality assessment, paediatric care in resource-limited settings or practical experience from each study country. The DSMB meets electronically or in person at least every 6 months. SAEs related to study participation are monitored in realtime, are summarised and reported to study investigators, WHO and relevant institutional review boards (IRBs) within 24 hours of notification in India and 72 hours of notification in Tanzania. Both sites are required to report to WHO within 72 hours of notification. Frequencies and descriptions of SAEs are pooled each month by the data management team at WHO and circulated to investigators, DSMB and IRBs. When more than half of the person-time is accrued in the study, the DSMB will review an interim data analysis by arm to determine whether stopping boundaries have been crossed.

Auditing

The sites are responsible for internal quality checks. WHO coordinators conduct at least one or two site monitoring visits per year to each site.

Discussion

Zinc supplementation in children during episodes of diarrhoea results in several advantages as described earlier.[1 7 12] Dose recommendations from WHO/Unicef were primarily based on previous clinical studies of zinc treatment of diarrhoea that used these amounts of zinc. Moreover, because zinc is absorbed less efficiently during diarrhoea,[17] it has been assumed that the therapeutic dose for treatment of diarrhoea should be substantially greater than the RDA for zinc (3–5 mg/day for children aged <5 years), depending on bioavailability from the usual diet.[9] Therefore, ZTDT was designed to examine the optimal therapeutic dose of zinc supplementation for children aged under 5 with acute diarrhoea in a south Asian country and a sub-Saharan African country. A recent search of clinicaltrials.gov found no other clinical trials that are determining an optimal zinc therapeutic dose for children with acute diarrhoea. An additionalsearch on PubMed, Google Scholar and other search engines was carried out where it was noted that one study was found to determine the optimal dose of zinc supplementation in diarrhoea.[18] The double-blind randomised trial was carried out using 10 or 20 mg zinc sulfate versus placebo. No difference in duration

nor frequency of diarrhoea was noted. However, this study was carried out in Switzerland, where the epidemiology of acute childhood diarrhoea is different than in India or Tanzania. As a result, ZTDT will likely be the first randomised trial in low-resource settings to determine the optimal dose of therapeutic zinc in children with acute diarrhoea.

Overall, the ZTDT study will measure important clinical outcomes (outcome of diarrhoea on growth, frequency of vomiting) in a large number of children under 5 years being treated for acute diarrhoea in India and Tanzania. It will also provide evidence for the effect of zinc on plasma zinc concentrations over several days after supplementation, the effect of zinc on post-illness outcomes as well as the acceptability of zinc supplementation. The trial results will be communicated in academic journals and at the national and local levels in Tanzania and India through policy briefs and dissemination meetings. The results of ZTDT will likely be generalisable to children under 5 years with acute diarrhoea in similar resource-limited settings and as such will have global relevance. The findings will be an important consideration in any further revision of the WHO technical guidelines on diarrhoea case management.

Author affiliations

1Department of Pediatrics, Muhimbili University of Health and Allied Sciences, Dar es Salaam, United Republic of Tanzania
2Centre for Public Health Kinetics (CPHK), New Delhi, Delhi, India
3Boston Children's Hospital, Boston, MA
4Harvard University T H Chan School of Public Health, Boston, Massachusetts, USA
5Division of Gastroenterology, Hepatology and Nutrition, Boston Children’s Hospital, Boston, MA
6Department of Nutrition, Harvard TH Chan School of Public Health, Boston, MA
7Tampereen yliopisto, Tampere, Finland
8Organisation mondiale de la Sante, Geneva, Switzerland

Acknowledgements

The authors express their gratitude to the external scientific reviewers of the proposal, for their valuable inputs, which led to important improvements in the study protocol. The authors appreciate the willingness of the DSMB members Dr William MacLeod (Chair), Dr Mario Gheri, Dr Godwin D Ndossi, Professor Rosalind Gibson and Professor Nita Bhandari to provide external study oversight. Additionally authors would like to acknowledge the India core field, data management and lab team: Dr. Pratibha Dixit, Dr. Aishwarya Chauhan, Vishi Saxena, Sanjay Kaushik, Vinod, Kumar, Manoj Kumar, Anita Rani and Arvind Sharma and Tanzanian study core team: Dr. Kristina Lugangira, Dr. Abraham Samma, Sr. Juliana Mghamba, Sr. Veneranda Ndesangia, Upendo Kibwana, Cecilia Msemwa, Zachariah Mtulo, Salmin Baleche and all the site staff for their support and hard-work in setting up of field and laboratory systems, piloting testing and SOP development.

Contributors

All the authors participated in conceptualisation, design, sample size calculations and writing of the analysis plan for the study. Two workshops held at WHO to develop joint harmonised SOP and CRFs for the study in which all the authors contributed. SSS and PD prepared the first draft of the paper. All authors contributed to revisions of the manuscript and contributed to the revision of the final draft of the manuscript. All the authors have read and approved the final manuscript.

Funding

This study is funded by Bill and Melinda Gates Foundation (grant number: OPP1135491) (see online supplementary additional file 5).

Disclaimer

The funder has no role in study design, collection, management, analysis and interpretation of data; writing of the report and decision to submit for publication.

Competing interests

None declared.

Patient consent for publication

Not required.

Ethics approval

The ZTDT protocol has been approved by WHO ethics committee (reference no. ERC.0002738), Boston Children's Hospital IRB (reference no .IRB-P00024269), Tanzania Food and Drug Authority (reference no. TFDA0016/CTR/0015/03), the Tanzanian National Institute of Medical Research (reference no. NIMR/HQ/R.8a/Vol.IX/2333) and Muhimbili University of Health and Allied Sciences, Dar es Salaam (reference no. 2016-10-31/AEC/Vol.XI/314) and IEC of Subharti Medical College & Hospital, Meerut, Uttar Pradesh, India (reference no. SMC/EC/2016/84) (see online supplementary additional file 4). All participants will be asked to provide written informed consents before trial enrolment. Caregivers who consent for the trial will also be asked for consent to be escorted back to their home to register the address, to contact the participant and to be visited at home or followed at home if the participant misses the clinic visit, as well as to store and use all data and child blood samples for this study and future research studies.

Provenance and peer review

Not commissioned; internally peer reviewed.

Data sharing statement

Full protocol is being published in a peer-reviewed open access journal. Additional information about the protocol/SOPs will be available on request. In addition, a commitment is made by the investigators: 1) to use data for research purposes and not to identify individual participants and 2) to use appropriate technology to secure the data. Consistent with WHO policies and practice, each site owns the data they generate from the national populations they serve. Sites voluntarily agree to share the data with WHO as the ZTDT Coordinating Body and share the final 'frozen data set' with BMGF for their internal use.

Author note

CD was supported in part by NIH grants K24DK104676 and 2P30 DK 040561.

Open access

This is an open access article distributed in accordance with the Creative Commons Attribution Non Commercial (CC BY-NC 4.0) license, which permits others to distribute, remix, adapt, build upon this work non-commercially, and license their derivative works on different terms, provided the original work is properly cited, appropriate credit is given, any changes made indicated, and the use is non-commercial. See: http:// creativecommons. org/ licenses/ by- nc/ 4. 0/.

References

1. Aggarwal R, Sentz J, Miller MA. Role of zinc administration in prevention of childhood diarrhea and respiratory illnesses: a metaanalysis. *Pediatrics* 2007;119:1120–30.
2. Bhatnagar S, Natchu UC. Zinc in child health and disease. *Indian J Pediatr* 2004;71:991–5.
3. Lukacik M, Thomas RL, Aranda JV. A meta-analysis of the effects of oral zinc in the treatment of acute and persistent diarrhea. *Pediatrics* 2008;121:326–36.
4. Prasad AS. Discovery of human zinc deficiency and studies in an experimental human model. *Am J Clin Nutr* 1991;53:403–12.
5. Haider BA, Bhutta ZA. The effect of therapeutic zinc supplementation among young children with selected infections: a review of the evidence. *Food Nutr Bull* 2009;30:S41–59.
6. Patel A, Mamtani M, Dibley MJ, *et al*. Therapeutic value of zinc supplementation in acute and persistent diarrhea: a systematic review. *PLoS One* 2010;5:e10386.
7. Lazzerini M, Wanzira H. Oral zinc for treating diarrhoea in children. *Cochrane Database Syst Rev* 2016;20:CD005436.
8. World Health Organization, Department of Child and Adolescent Health and Development. *The treatment of diarrhoea: a manual for physicians and other senior health workers*. Geneva: Dept. of Child and Adolescent Health and Development, World Health Organization, 2005.
9. Institute of Medicine (US) Panel on Micronutrients. *Dietary Reference Intakes for Vitamin A, Vitamin K, Arsenic, Boron, Chromium, Copper, Iodine, Iron, Manganese, Molybdenum, Nickel, Silicon, Vanadium, and Zinc*. Washington (DC): National Academies Press (US), 2001.
10. Strand TA, Chandyo RK, Bahl R, *et al*. Effectiveness and efficacy of zinc for the treatment of acute diarrhea in young children. *Pediatrics* 2002;109:898–903.
11. Bahl R, Bhandari N, Saksena M, *et al*. Efficacy of zinc-fortified oral rehydration solution in 6- to 35-month-old children with acute diarrhea. *J Pediatr* 2002;141:677–82.
12. Sazawal S, Black RE, Bhan MK, *et al*. Zinc supplementation in young children with acute diarrhea in India. *N Engl J Med* 1995;333:839–44.
13. World Health Organization, Department of Maternal N Child and Adolescent Health, World Health Organization. Revised WHO classification and treatment of pneumonia in children at health facilities: evidence summaries. 2014 http:// apps. who. int/ iris/ bitstream/ 10665/ 137319/ 1/ 9789241507813_ eng. pdf? ua=1 (Accessed 21st July 2018).
14. IZiNCG Technical Briefs. IZiNCG. http://www. izincg. org/ technicalbriefs/ (Accessed 21st July 2018).
15. Mauri L, D'Agostino RB. Challenges in the Design and Interpretation of Noninferiority Trials. *N Engl J Med* 2017;377:1357–67.
16. WHO/UNICEF Joint Monitoring Programme (JMP) for Water Supply and Sanitation. Improved and unimproved water and sanitation

facilities. 2014 http://www. wssinfo. org/ definitions- methods/ watsancategories/
17. Castillo-Duran C, Heresi G, Fisberg M, *et al*. Controlled trial of zinc supplementation during recovery from malnutrition: effects on growth and immune function. *Am J Clin Nutr* 1987;45:602–8.
18. Crisinel PA, Verga ME, Kouame KS, *et al*. Demonstration of the effectiveness of zinc in diarrhoea of children living in Switzerland. *Eur J Pediatr* 2015;174:1061–7. bmjpo: first published as 10.1136/bmjpo-2019-000460 on 24 April 2019. Downloaded from http://bmjpaedsopen.bmj.com/ on February 19, 2020 by guest. Protected by copyright.

Supplementary Figures

Supplementary Figure 1. Forest plot for differences in proportion of children with diarrhea duration > 5 days. Absolute differences (■) and upper 95% confidence intervals (•) between low dose and standard dose arms. The non-inferiority margin was defined a priori at 4.0%. In none of the performed analyses was the non-inferiority margin exceeded.

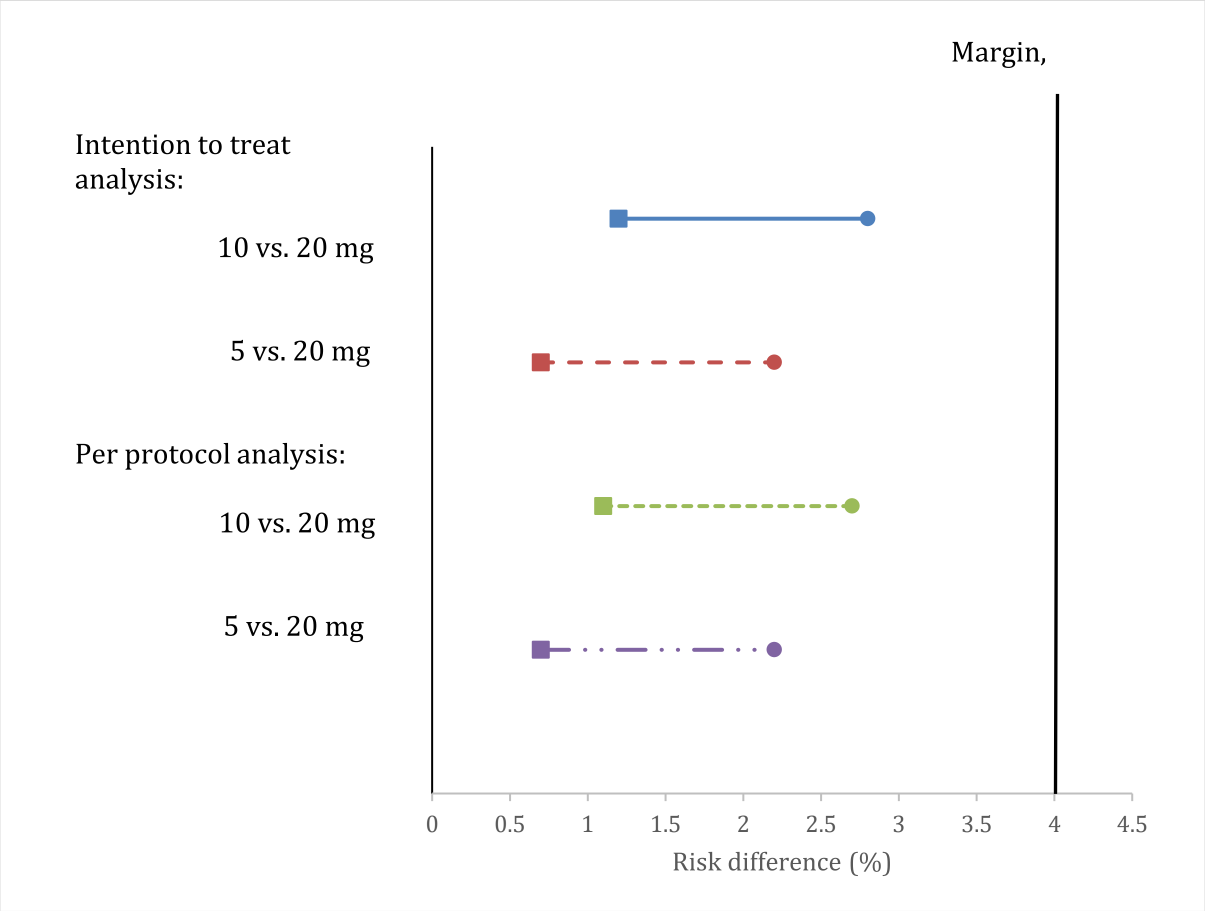

Supplementary Figure 2. Forest plot for differences in mean number of total loose or watery stools. Absolute differences (■) and upper 95% confidence intervals (•) between low dose and standard dose arms. The non-inferiority margin was defined a priori at 2 stools. In none of the performed analyses was the non-inferiority margin exceeded.

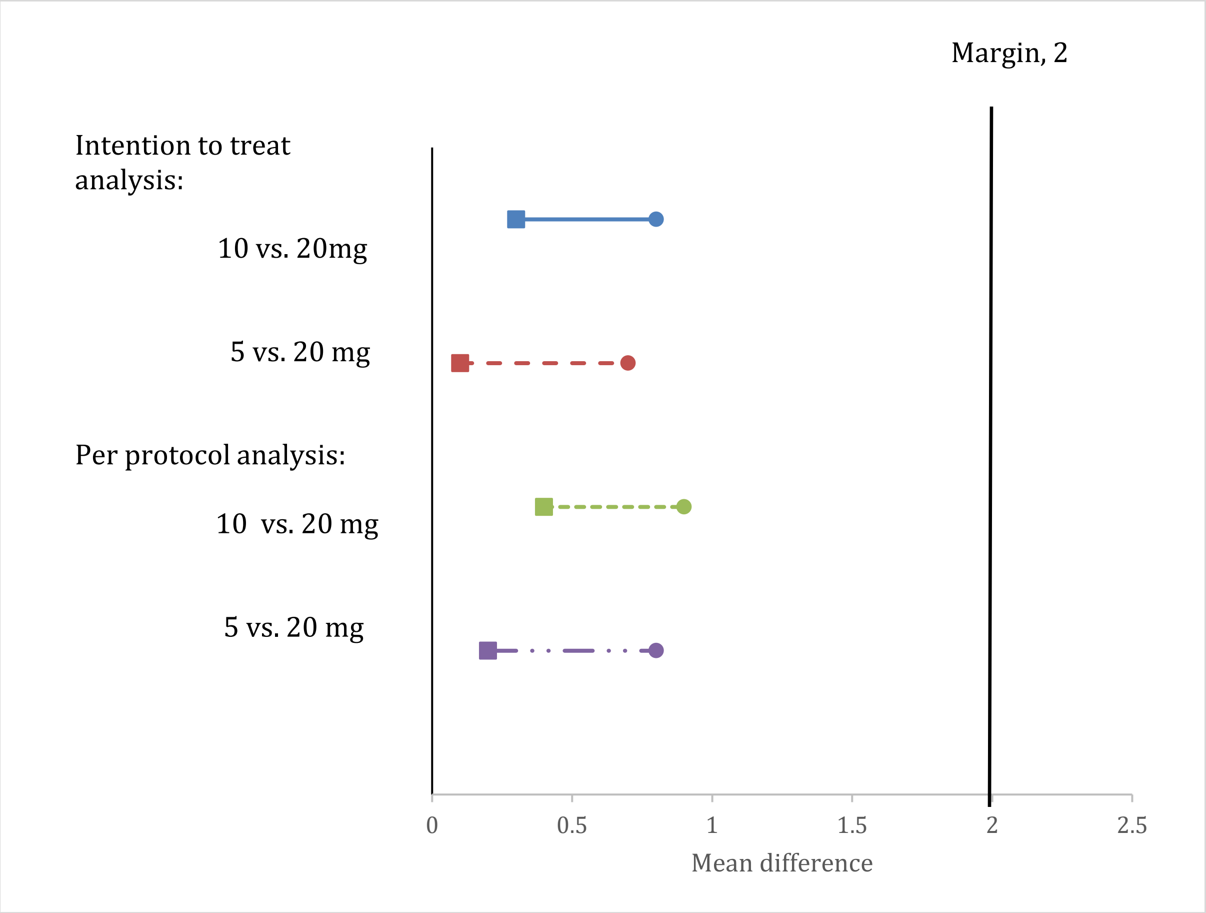

Supplementary Figure 3. Kaplan-Meier Curve for time to initial diarrhea episode recovery by zinc supplementation dose

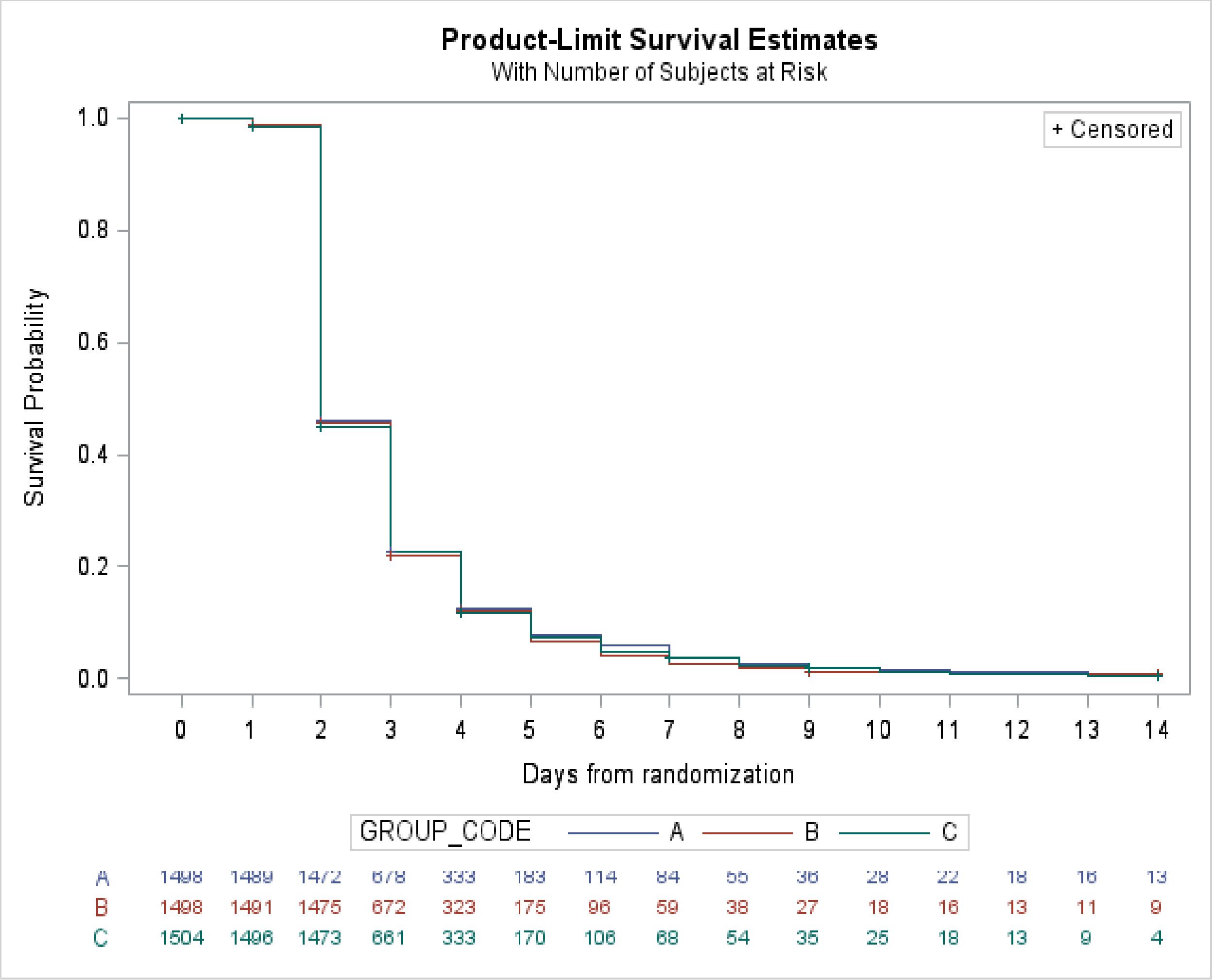

Supplementary Tables

Supplementary Table 1. Baseline characteristics of the India trial population stratified by zinc supplementation dose (n=2250)

	5 mg (N=751) Mean ± SD or n (%)	10 mg (N=749) Mean ± SD or n (%)	20 mg (N=750) Mean ± SD or n (%)
Maternal and household characteristics			
Maternal age, years	26.4 ± 4.2	26.4 ± 4.0	26.3 ± 3.9
Maternal education, years	6.6 ± 5.0	6.5 ± 4.9	6.8 ± 4.7
No maternal education	219 (29.2)	214 (28.6)	188 (25.1)
Improved water	736 (98.7)	739 (98.9)	741 (99.1)
Improved sanitation	743 (99.6)	747 (100.0)	747 (99.9)
Household wealth above median[1]	379 (50.8)	370 (49.5)	371 (49.6)
Child characteristics			
Age at randomization (months)	26.6 ± 16.2	26.4 ± 16.4	27.0 ± 17.0
Age at randomization			
6 to <12 months	171 (22.8)	180 (24.0)	193 (25.7)
12 to <24 months	207 (27.6)	199 (26.6)	186 (24.8)
24 to <60 months	373 (49.7)	370 (49.4)	371 (49.5)
Female	354 (47.1)	374 (49.9)	362 (48.3)

Breastfeeding day prior to enrollment	422 (56.6)	406 (54.4)	402 (53.8)
Rotavirus vaccination	1 (0.1)	0 (0.0)	2 (0.3)
Duration of diarrhea before enrollment			
≤ 24 hours	34 (4.5)	28 (3.7)	33 (4.4)
25 to 48 hours	697 (92.8)	695 (92.8)	686 (91.5)
49 to <72 hours	20 (2.7)	26 (3.5)	31 (4.1)
Number of loose or watery stools in the 24 hours before enrollment	6.4 ± 2.1	6.4 ± 2.2	6.4 ± 2.1
Dysentery	11 (1.5)	17 (2.3)	19 (2.5)
Some dehydration	3 (0.4)	0 (0.0)	3 (0.4)
Axillary temperature >38°C	13 (1.7)	9 (1.2)	10 (1.3)
Cough or difficulty breathing	279 (37.2)	312 (41.7)	281 (37.5)
Observed respiratory rate >40 bpm	12 (1.6)	10 (1.3)	6 (0.8)
Prior antibiotic use	32 (2.1)	26 (1.7)	38 (2.5)
Height (cm)	81.5 ± 12.0	81.4 ± 12.2	82.0 ± 13.0
Weight (kg)	10.1 ± 2.7	10.1 ± 2.7	10.2 ± 3.0
MUAC (cm)	13.7 ± 1.2	13.7 ± 1.1	13.8 ± 1.2
Mean LAZ/HAZ	-1.7 ± 1.2	-1.6 ± 1.1	-1.6 ± 1.2
Mean WLZ/WHZ	-0.9 ± 0.9	-0.9 ± 0.9	-0.9 ± 0.9
Mean WAZ	-1.6 ± 1.0	-1.5 ± 0.9	-1.5 ± 1.0
Mean MUACZ	-1.3 ± 0.9	-1.2 ± 0.9	-1.2 ± 0.9

Stunted (LAZ/HAZ < -2)	307 (40.9)	258 (34.5)	257 (34.3)
Wasted (WLZ/WHZ < -2	89 (11.9)	84 (11.2)	93 (12.4)
Underweight (WAZ < -2)	270 (36.0)	234 (31.2)	234 (31.2)
Zinc dose (mg/kg body weight)	0.53 ± 0.14	1.07 ± 0.28	2.13 ± 0.62
Plasma zinc concentration (μg/dL)[2]	80.7 ± 22.1	81.8 ± 22.1	82.9 ± 22.9
Plasma zinc concentration <65 μg/dL[3]	58 (24.9)	48 (19.9)	61 (23.2)

[1]Country-specific household wealth index was constructed using a principal component analysis of household ownership, household assets, drinking water source, and sanitation

[2] Plasma zinc concentrations were assessed in a random sample of 33% of participants at baseline at each site

Supplementary Table 2. Baseline characteristics of Tanzania trial population stratified by zinc supplementation dose (n=2250)

	5 mg (N=753) Mean ± SD or n (%)	10 mg (N=749) Mean ± SD or n (%)	20 mg (N=748) Mean ± SD or n (%)
Maternal and household characteristics			
Maternal age, years	27.0 ± 5.6	27.2 ± 5.7	27.5 ± 5.9
Maternal education, years	8.0 ± 3.0	7.7 ± 2.9	7.8 ± 3.0
No maternal education	38 (5.2)	40 (5.4)	42 (5.7)
Improved water	749 (99.5)	746 (99.6)	744 (99.5)
Improved sanitation	743 (98.7)	741 (98.9)	742 (99.2)
Household wealth above median[1]	381 (50.6)	368 (49.1)	401 (53.6)
Child characteristics			
Age at randomization (months)	19.4± 12.3	19.0 ± 11.3	19.4 ± 12.2
Age at randomization			
6 to <12 months	241 (32.0)	230 (30.7)	241 (32.2)
12 to <24 months	292 (38.8)	303 (40.5)	290 (38.8)
24 to <60 months	220 (29.2)	216 (28.8)	217 (29.0)
Female	357 (47.4)	351 (46.9)	357 (47.7)

Breastfeeding day prior to enrollment	454 (60.3)	451 (60.2)	451 (60.3)
Rotavirus vaccination	748 (99.5)	746 (100.0)	741 (98.9)
Duration of diarrhea before enrollment			
≤ 24 hours	24 (3.2)	20 (2.7)	26 (3.5)
25 to 48 hours	562 (74.6)	529 (70.6)	545 (72.9)
49 to <72 hours	167 (22.2)	200 (26.7)	177 (23.7)
Number of loose or watery stools in the 24 hours before enrollment	6.4 ± 2.1	6.4 ± 2.2	6.4 ± 2.1
Dysentery	43 (5.7)	45 (6.0)	32 (4.3)
Some dehydration	29 (3.9)	11 (1.5)	10 (1.3)
Axillary temperature >38°C	35 (4.7)	31 (4.1)	24 (3.2)
Cough or difficulty breathing	126 (16.7)	143 (19.1)	126 (16.8)
Observed respiratory rate >40 bpm	74 (9.8)	78 (10.4)	75 (10.0)
Prior antibiotic use	28 (3.7)	31 (4.1)	20 (2.7)
Height (cm)	78.3 ± 9.6	77.9 ± 9.4	78.2 ± 9.9
Weight (kg)	9.8 ± 2.2	9.6 ± 2.1	9.8 ± 2.3
MUAC (cm)	14.5 ± 1.1	14.3 ± 1.1	14.4 ± 1.1
Mean LAZ/HAZ	-0.9 ± 1.1	-1.0 ± 1.0	-1.0 ± 1.1
Mean WLZ/WHZ	-0.4 ± 1.1	-0.5 ± 1.1	-0.4 ± 1.1
Mean WAZ	-0.8 ± 1.0	-0.9 ± 1.0	-0.8 ± 1.1
Mean MUACZ	-0.3 ± 0.9	-0.4 ± 0.9	-0.4 ± 0.9

Stunted (LAZ/HAZ < -2)	117 (15.5)	124 (16.6)	125 (16.7)
Wasted (WLZ/WHZ < -2	42 (5.6)	51 (6.8)	48 (6.4)
Underweight (WAZ < -2)	83 (11.0)	114 (15.2)	99 (13.2)
Zinc dose (mg/kg body weight)	0.53 ± 0.11	1.08 ± 0.22	2.15 ± 0.45
Plasma zinc concentration (µg/dL)[2]	60.9 ± 20.0	66.3 ± 23.3	60.7 ± 27.5
Plasma zinc concentration <65 µg/dL[3]	117 (57.6)	95 (49.7)	113 (63.5)

[1]Country-specific household wealth index was constructed using a principal component analysis of household ownership, household assets, drinking water source, and sanitation

[2] Plasma zinc concentrations were assessed in a random sample of 33% of participants at baseline at each site

Supplementary Table 3. Subgroup analysis for results on proportion of children with diarrhea >5 days (primary outcome 1)

Potential effect modifier (baseline characteristic)	RD 95% CI (5mg vs 20mg)	RD 95% CI (10mg vs 20mg)
Study site		
India	0.0%(-2.7%, 2.8%)	1.1%(-1.8%, 3.9%)
Tanzania	1.3%(-1.0%, 3.7%)	1.4% (-1.0%, 3.7%)
Age group		
<12 month	-1.0%(-5.5%,3.5%)	0.7%(-3.9%,5.4%)
≥12 months	1.5%(-0.3%,3.3%)	1.6%(-0.2%,3.4%)
Sex		
Male	0.6%(-2.0%,3.2%)	2.0%(-0.7%,4.8%)
Female	0.8%(-1.8%,3.3%)	0.3%(-2.2%,2.8%)
Rotavirus vaccine received		
No	0.0%(-2.8%,2.8%)	1.0%(-1.9%,3.8%)
yes	1.4%(-1.0%,3.7%)	1.4%(-0.9%,3.8%)
Maternal HIV status		
No	1.3%(-1.1%,3.7%)	1.7%(-0.7%,4.2%)
Yes	n.e.	n.e.
Dysentery		
No	1.1%(-0.8%,2.9%)	1.3%(-0.6%,3.1%)

Yes	-8.7%(-20.3%,2.8%)	0.2%(-12.9%,13.4%)
Dehydration		
no	0.5%(-1.3%,2.3%)	1.1%(-0.7%,3.0%)
Yes	8.4%(-11.0%,27.9%)	14.5%(-16.3%,45.3%)
Axillary temp >38°C		
No	0.6%(-1.2%,2.4%)	1.4%(-0.5%,3.2%)
yes	1.5%(-15.1%,18.2%)	-5.2%(-20.5%,10.2%)
Respiratory rate > 40 /min		
no	0.4%(-1.5%,2.3%)	0.8%(-1.1%,2.7%)
yes	5.0%(-2.3%,12.2%)	8.9%(0.7%,17.2%)
Recent antibiotic use		
No	0.6%(-1.3%,2.4%)	1.2%(-0.6%,3.1%)
Yes	5.2%(-7.7%,18.1%)	1.1%(-9.6%,11.8%)
Breastfed		
No	1.8%(-0.2%,3.9%)	2.2%(0.1%,4.2%)
Yes	-0.8%(-4.1%,2.4%)	-0.1%(-3.4%,3.3%)
Stunted		
No	1.2%(-1.0%,3.3%)	1.5%(-0.6%,3.7%)
Yes	-0.7%(-4.2%,2.8%)	0.3%(-3.4%,4.0%)
Underweight		
No	0.4%(-1.7%,2.4%)	1.5%(-0.6%,3.6%)

Yes	1.6%(-2.5%,5.8%)	0.2%(-3.8%,4.1%)
Wasted		
No	0.8%(-1.1%,2.7%)	1.4%(-0.6%,3.3%)
yes	-1.0%(-7.3%,5.3%)	-0.5%(-6.8%,5.9%)
MUAC less than 125 mm		
No	1.4%(-0.5%,3.2%)	1.9%(0.1%,3.8%)
Yes	-7.6%(-15.9%,0.7%)	-7.3%(-15.5%,1.0%)
Family wealth < median		
No	0.2%(-2.2%,2.6%)	2.6%(-0.02%,5.2%)
Yes	1.1%(-1.6%,3.9%)	-0.0%(-2.8%,2.5%)
Maternal education years < 8		
No	1.5%(-1.4%,4.4%)	2.1%(-0.9%,5.1%)
Yes	0.1%(-2.2%,2.5%)	0.7%(-1.6%,3.1%)
HH water quality (improved vs. unimproved)		
No	n.e.	n.e.
Yes	0.6%(-1.2%,2.5%)	1.1%(-0.8%,2.9%)
HH sanitation quality (improved vs. unimproved)		
No	0.6%(-1.2%,2.5%)	1.1%(-0.7%,2.9%)
Yes	0.7%(-1.1%,2.5%)	1.2%(-0.6%,3.1%)
Baseline plasma zinc <65 µg/dL		

No	-2.9%(-7.4%,1.6%)	-3.4%(-7.8%,0.9%)
Yes	4.5%(-1.2%,10.2%)	4.9%(-1.3%,11.1%)

n.e.: Not estimable due to no child having diarrhea duration >5 days in the subgroup

Supplementary Table 4. Subgroup analysis for results on total number of stools (primary outcome 2)

**Potential effect modifier (baseline characteristic)**	**MD 95% CI (5mg vs 20mg)**	**MD 95% CI (10mg vs 20mg)**
Study site		
India	0.1 (-1.0, 1.1)	0.3 (-0.7, 1.4)
Tanzania	0.1 (-0.6, 0.9)	0.2 (-0.6, 1.0)
Age group		
<12 month	0.1(-1.6, 1.8)	0.8(-0.9, 2.6)
≥12 months	0.2(-0.4, 0.7)	0.1(-0.5, 0.7)
Sex		
Male	-0.2(-1.1, 0.8)	0.3(-0.6, 1.3)
Female	0.3(-0.5, 1.2)	0.2 (-0.7, 1.0)
Rotavirus vaccine received		
No	0.0(-1.0,1.1)	0.3(-0.8,1.3)
yes	0.1(-0.6,0.9)	0.2(-0.5,1.0)
Maternal HIV status		
No	0.1(-0.6,0.9)	0.3(-0.5,1.0)
Yes	0.2(-3.8,4.3)	-2.0(-6.4,2.4)
Dysentery		
No	0.1(-0.6,0.7)	0.1(-0.5,0.8)

Yes	0.3(-3.9,4.5)	3.5(-0.6,7.5)
Dehydration		
no	0.0(-0.6,0.7)	0.2(-0.4,0.9)
Yes	2.7(-4.9,10.4)	7.6(-2.5,17.6)
Axillary temp >38°C		
No	0.1(-0.6,0.7)	0.3(-0.3,1.0)
yes	-0.8(-5.4,3.7)	-2.0(-6.6,2.7)
Respiratory rate > 40 /min		
no	-0.0(-0.7,0.6)	0.1(-0.6,0.8)
yes	1.7(-0.8,4.1)	3.1(0.7,5.6)
Recent antibiotic use		
No	0.1(-0.6,0.7)	0.2(-0.4,0.9)
Yes	1.6(-2.5,5.7)	1.4(-2.5,5.4)
Breastfed		
No	0.5(-0.4,1.2)	0.5(-0.2,1.3)
Yes	-0.4(-1.5,0.8)	-0.1(-1.3,1.0)
Stunted		
No	0.1(-0.7,0.8)	0.2(-0.5,1.0)
Yes	0.1(-1.1,1.3)	0.4(-0.9,1.6)
Underweight		
No	-0.1(-0.8,0.7)	0.3(-0.4,1.0)

Yes	0.5(-1.0,1.9)	0.1(-1.4,1.6)
Wasted		
No	0.1(-0.6,0.7)	0.2(-0.4,0.9)
yes	0.1(-2.9,3.0)	0.9(-2.0,3.8)
MUAC less than 125 mm		
No	0.1(-0.5,0.8)	0.4(-0.3,1.0)
Yes	-0.9(-3.8,2.0)	-1.4(-4.3,1.5)
Family wealth < median		
No	0.0(-1.0,1.0)	-0.1(-1.1,0.9)
Yes	0.2(-0.6,1.0)	0.6(-0.2,1.4)
Maternal education years < 8		
No	0.1(-0.9,1.1)	0.4(-0.6,1.5)
Yes	0.1(-0.7,0.9)	0.2(-0.6,1.0)
HH water quality (improved vs. unimproved)		
No	-0.7(-6.6,5.2)	-0.5(-7.0,6.0)
Yes	0.1(-0.6,0.7)	0.3(-0.4,0.9)
HH sanitation quality (improved vs. unimproved)		
No	0.6(-5.4,6.7)	0.5(-6.0,6.9)
Yes	0.1(-0.6,0.7)	0.3(-0.4,0.9)
Baseline plasma zinc <65 μg/dL		

No	-0.7(-2.4,0.9)	-1.1(-2.7,0.5)
Yes	1.4(-0.6,3.4)	1.3(-0.8,3.3)

Supplementary Table 5. Subgroup analysis for results on vomiting (primary outcome 3)

Potential effect modifier (baseline characteristic)	RR 95% CI (5mg vs 20mg))	RR 95% CI (10mg vs 20mg)
Study site		
India	0.30 (0.20, 0.44)	0.52 (0.38, 0.72)
Tanzania	0.93 (0.77, 1.11)	0.96 (0.80, 1.14)
Age group		
<12 month	0.76 (0.61, 0.96)	0.72 (0.56, 0.91)
≥12 months	0.69 (0.55, 0.86)	0.89 (0.72, 1.09)
Sex		
Male	0.74 (0.59, 0.92)	0.74 (0.59, 0.93)
Female	0.68 (0.54, 0.87)	0.88 (0.70, 1.09)
Rotavirus vaccine received		
No	0.30 (0.20, 0.44)	0.52 (0.37, 0.71)
yes	0.93 (0.78, 1.11)	0.97 (0.81, 1.15)
Maternal HIV status		
No	0.94 (0.79, 1.13)	0.96 (0.80, 1.15)
Yes	1.32 (0.49, 3.58)	1.17 (0.38, 3.55)
Dysentery		
No	0.67 (0.57, 0.79)	0.79 (0.67, 0.93)

Yes	2.52 (1.07, 5.93)	1.51 (0.60, 3.80)
Dehydration		
no	0.69 (0.59, 0.82	0.80 (0.69, 0.94)
Yes	1.35 (0.44, 4.14)	1.18 (0.30, 4.72)
Axillary temp >38°C		
No	1.02 (0.42, 2.45)	1.21 (0.52, 2.84)
yes	0.70 (0.59, 0.83)	0.79 (0.68, 0.93)
Respiratory rate > 40 /min		
no	0.69 (0.58, 0.82)	0.79 (0.67, 0.93)
yes	0.99 (0.60, 1.62	0.97 (0.59, 1.60
Recent antibiotic use		
No	0.71 (0.60, 0.83)	0.80 (0.68, 0.93)
Yes	0.81 (0.38, 1.75)	0.91 (0.45, 1.85)
Breastfed		
No	0.63 (0.46, 0.85)	0.90 (0.69, 1.19)
Yes	0.76 (0.63, 0.92)	0.76 (0.63, 0.92)
Stunted		
No	0.79 (0.66, 0.95)	0.80 (0.67, 0.95)
Yes	0.47 (0.31, 0.70)	0.84 (0.60, 1.18)
Underweight		
No	0.78 (0.65, 0.93)	0.82 (0.69, 0.97)

Yes	0.45 (0.29, 0.69)	0.77 (0.54, 1.10)
Wasted		
No	0.72 (0.61, 0.85)	0.81 (0.69, 0.96)
yes	0.62 (0.34, 1.12	0.72 (0.42, 1.26)
MUAC less than 125 mm		
No	0.74 (0.63, 0.88)	0.84 (0.71, 0.99)
Yes	0.36 (0.18, 0.75)	0.47 (0.24, 0.90)
Family wealth < median		
No	0.59 (0.47, 0.74)	0.78 (0.64, 0.97)
Yes	0.88 (0.70, 1.12)	0.84 (0.66, 1.07)
Maternal education years < 8		
No	0.56 (0.43, 0.73)	0.79 (0.63, 1.01)
Yes	0.84 (0.68, 1.04)	0.81 (0.66, 1.01)
HH water quality (improved vs. unimproved)		
No	0.52 (0.11, 2.61)	1.33 (0.39, 4.62)
Yes	0.71 (0.61, 0.84)	0.80 (0.68, 0.94)
HH sanitation quality (improved vs. unimproved)		
No	1.62 (0.20, 12.78)	0.87 (0.07, 11.54)
Yes	0.71 (0.60, 0.83)	0.81 (0.69, 0.94)
Baseline plasma zinc <65 μg/dL		

No	0.67 (0.43, 1.04)	0.60 (0.39, 0.94)
Yes	0.60 (0.37, 0.97)	0.64 (0.39, 1.05)

Supplementary Table 6. Mean difference in plasma zinc concentrations (µg/dL) at baseline, Day 3, Day 7, Day 15, Day 21 and Day 30 for the 5 mg group and 10 mg group as compared to 20 mg group

	5 mg plasma samples (n)	5 mg group Plasma zinc Mean ± SD (µg/dL)	Mean difference* (µg/dL) 5 mg group as compared to 20 mg group (95% CI)	10 mg plasma samples (n)	10 mg group Plasma zinc Mean ± SD (µg/dL)	Mean difference* (µg/dL) 10 mg group as compared to 20 mg group (95% CI)	20 mg plasma samples (n)	20 mg Group Plasma zinc Mean ± SD (µg/dL)
Baseline [Day 1]	436	71.5 ± 23.4	-2.5 (-5.8, 0.9)	432	74.9 ± 23.9	1.0 (-2.4, 4.4)	441	74.0 ± 27.1
Day 3 [Day 3-5]	391	84.1 ± 31.1	-14.9 (-20.0, -9.8)	379	93.9 ± 42.1	-5.1 (-11.0, 0.8)	397	99.0 ± 41.5
Day 7 [Day 6-11]	408	85.1 ± 29.9	-19.0 (-24.6, -13.5)	419	92.3 ± 37.3	-10.8 (-16.7, -5.0)	403	104.1 ± 48.3
Day 15 [Day 12 - 18]	370	79.7 ± 25.9	-10.7 (-15.4, -6.1)	361	82.6 ± 27.8	-7.8 (-12.6, -3.0)	385	90.5 ± 38.0
Day 21 [Day 19-25]	392	74.3 ± 21.3	-2.0 (-5.1, 1.2)	383	77.6 ± 25.1	1.4 (-2.1, 4.8)	382	76.3 ± 23.3
Day 30 [Day 26-45]	409	73.6 ± 21.7	-3.8 (-7.0, -0.7)	434	74.4 ± 22.7	-3.0 (-6.2, 0.2)	410	77.4 ± 24.5

*Mean difference and confidence intervals estimated from linear mixed-effects model

References

1. Black R, Fontaine O, Lamberti L, et al. Drivers of the reduction in childhood diarrhea mortality 1980-2015 and interventions to eliminate preventable diarrhea deaths by 2030. Journal of global health 2019;9:020801.

2. WHO/UNICEF. Clinical management of acute diarrhoea. Geneva: WHO; 2004. Report No.: WHO/FCH/CAH 04.7.

3. Patel A, Mamtani M, Dibley MJ, Badhoniya N, Kulkarni H. Therapeutic value of zinc supplementation in acute and persistent diarrhea: a systematic review. PLoS One 2010;5:e10386.

4. Lukacik M, Thomas RL, Aranda JV. A Meta-analysis of the Effects of Oral Zinc in the Treatment of Acute and Persistent Diarrhea. Pediatrics 2008;121:326-36.

5. Brooks WA, Santosham M, Roy SK, et al. Efficacy of zinc in young infants with acute watery diarrhea. Am J Clin Nutr 2005;82:605-10.

6. Bhatnagar S, Bahl R, Sharma PK, Kumar GT, Saxena SK, Bhan MK. Zinc With Oral Rehydration Therapy Reduces Stool Output and Duration of Diarrhea in Hospitalized Children: A Randomized Controlled Trial. Journal of Pediatric Gastroenterology & Nutrition 2004;38:34-40.

7. Strand TA, Chandyo RK, Bahl R, et al. Effectiveness and Efficacy of Zinc for the Treatment of Acute Diarrhea in Young Children. Pediatrics 2002;109:898-903.

8. Zinc Investigators Collaborative Group. Therapeutic effects of oral zinc in acute and persistent diarrhea in children in developing countries: pooled analysis of randomized controlled trials. Am J Clin Nutr 2000;72:1516-22.

9. Sazawal S, Black RE, Bhan MK, Bhandari N, Sinha A, Jalla S. Zinc supplementation in young children with acute diarrhea in India. N Engl J Med 1995;333:839-44.

10. Standing Committee on the Scientific Evaluation of Dietary Reference Intakes - Food and Nutrition Board - Institute of Medicine. Dietary Reference Intakes for Vitamin A, Vitamin K, Arsenic, Boron, Chromium, Copper, Iodine, Iron, Manganese, Molybdenum, Nickel, Silicon, Vanadium, and Zinc. Washington, DC: National Academy Press; 2002.

11. de Brito NJ, Rocha ED, de Araujo Silva A, et al. Oral zinc supplementation decreases the serum iron concentration in healthy schoolchildren: a pilot study. Nutrients 2014;6:3460-73.

12. Carter RC, Kupka R, Manji K, et al. Zinc and multivitamin supplementation have contrasting effects on infant iron status: a randomized, double-blind, placebo-controlled clinical trial. Eur J Clin Nutr 2018;72:130-5.

13. Simon SR, Branda RF, Tindle BF, Burns SL. Copper deficiency and sideroblastic anemia associated with zinc ingestion. Am J Hematol 1988;28:181-3.

14. Larson CP, Hoque A, Larson C, Khan A, Saha U. Initiation of Zinc Treatment for Acute Childhood Diarrhoea and Risk for Vomiting orRegurgitation: A Randomized, Double-blind, Placebo-controlled Trial. J Health Popul Nutr 2005;23:311-9.

15. Lazzerini M, Ronfani L. Oral zinc for treating diarrhoea in children. Cochrane database of systematic reviews 2008:CD005436.

16. Somji SS, Dhingra P, Dhingra U, et al. Effect of dose reduction of supplemental zinc for childhood diarrhoea: study protocol for a double-masked, randomised controlled trial in India and Tanzania. BMJ Paediatr Open 2019;3:e000460.

17. International Zinc Nutrition Consultative Group. Assessing population zinc status with serum zinc concentration. IZINCG technical brief no. 22012.

18. Bahl R, Bhandari N, Saksena M, et al. Efficacy of zinc-fortified oral rehydration solution in 6- to 35-month-old children with acute diarrhea. J Pediatr 2002;141:677-82.

19. Mauri L, D'Agostino RB, Sr. Challenges in the Design and Interpretation of Noninferiority Trials. N Engl J Med 2017;377:1357-67.

20. Spiegelman D, Hertzmark E. Easy SAS calculations for risk or prevalence ratios and differences. Am J Epidemiol 2005;162:199-200.

21. Pedroza C, Thanh Truong VT. Performance of models for estimating absolute risk difference in multicenter trials with binary outcome. BMC Med Res Methodol 2016;16:113.

22. WHO. Handbook: IMCI integrated management of childhood illness. Geneva, Switzerland: World Health Organization; 2005.

23. Olofin I, McDonald CM, Ezzati M, et al. Associations of suboptimal growth with all-cause and cause-specific mortality in children under five years: a pooled analysis of ten prospective studies. PLoS One 2013;8:e64636.

24. Santosham M, Chandran A, Fitzwater S, Fischer-Walker C, Baqui AH, Black R. Progress and barriers for the control of diarrhoeal disease. Lancet 2010;376:63-7.

25. Black R, et al. Control of Childhood Diarrhea Mortality 1980-2015 and Interventions to Eliminate Preventable Diarrhea Deaths by 2030. The Lancet Global health 2019.

26. Gill CJ, Young M, Schroder K, et al. Bottlenecks, barriers, and solutions: results from multicountry consultations focused on reduction of childhood pneumonia and diarrhoea deaths. The Lancet 2013;381:1487-98.

27. Larson CP, Koehlmoos TP, Sack DA, Scaling Up of Zinc for Young Children Project T. Scaling up zinc treatment of childhood diarrhoea in Bangladesh: theoretical and practical considerations guiding the SUZY Project. Health Policy Plan 2012;27:102-14.

28. Chandola TR, Taneja S, Goyal N, et al. Descriptive epidemiology of rotavirus infection in a community in North India. Epidemiol Infect 2013;141:2094-100. doi: 10.1017/S0950268812002762. Epub 2013 Jan 8.

29. Becker-Dreps S, Bucardo F, Vilchez S, et al. Etiology of childhood diarrhea after rotavirus vaccine introduction: a prospective, population-based study in Nicaragua. Pediatr Infect Dis J 2014;33:1156-63.

30. Patel AB, Dibley MJ, Mamtani M, Badhoniya N, Kulkarni H. Influence of zinc supplementation in acute diarrhea differs by the isolated organism. Int J Pediatr 2010;2010:671587.

31. Hoque KM, Binder HJ. Zinc in the treatment of acute diarrhea: current status and assessment. Gastroenterology 2006;130:2201-5.

32. Rahman MJ, Sarker P, Roy SK, et al. Effects of zinc supplementation as adjunct therapy on the systemic immune responses in shigellosis. Am J Clin Nutr 2005;81:495-502.

33. Hoque KM, Rajendran VM, Binder HJ. Zinc inhibits cAMP-stimulated Cl secretion via basolateral K-channel blockade in rat ileum. Am J Physiol Gastrointest Liver Physiol 2005;288:G956-63.
